# High‐Performance Zinc–Bromine Rechargeable Batteries Enabled by In‐Situ Formed Solid Electrolyte Interphase

**DOI:** 10.1002/advs.202508646

**Published:** 2025-09-29

**Authors:** Norah S. Alghamdi, Xiyue Peng, Xingchen Yang, Shengyong Gao, Yongxin Huang, Shuangbin Zhang, Tong'en Lin, Cheng Zhang, Ian R. Gentle, Lianzhou Wang, Bin Luo

**Affiliations:** ^1^ Australian Institute for Bioengineering and Nanotechnology The University of Queensland Brisbane QLD 4072 Australia; ^2^ School of Chemistry and Molecular Biosciences The University of Queensland Brisbane QLD 4072 Australia; ^3^ Department of Chemistry Faculty of Science Imam Mohammad Ibn Saud Islamic University (IMSIU) Riyadh 11564 Saudi Arabia; ^4^ Department of Applied Biology and Chemical Technology Faculty of Science The Hong Kong Polytechnic University Hong Kong SAR P. R. China; ^5^ School of Chemical Engineering The University of Queensland Brisbane QLD 4072 Australia

**Keywords:** aqueous electrolyte, hydrogen evolution reaction, solid electrolyte interphase, zinc–bromine batteries, zinc dendrites

## Abstract

Aqueous zinc–bromine batteries (ZBBs) are promising candidates for renewable energy storage, offering advantages over lithium‐ion batteries. However, their widespread adoption is hindered by challenges such as zinc dendrite formation and water decomposition, which lead to short circuits, electrode degradation and reduced cycle life. Therefore, this study presents a facile strategy for in‐situ construction of a fluorinated solid electrolyte interphase (SEI) formed via coating graphite current collectors with a lubricant hydrophobic perfluoropolyether interlayer. During the initial charging process, a fluoride‐rich SEI layer forms to regulate Zn nucleation and suppress dendrite growth. This SEI promotes uniform zinc deposition and inhibits hydrogen evolution by limiting water access to the electrode surface, thereby enhancing cycle life and energy efficiency. As a result, ZBBs incorporating this SEI exhibit a substantial reduction in potential hysteresis from 285 to 60 mV, deliver an energy density of nearly 20 Wh L^−1^ and an areal capacity of 10.7 mAh cm^−2^, and maintain >79% energy efficiency over 1000 cycles. This work offers a scalable approach to achieving high‐performance ZBBs, advancing the development of next‐generation anode‐free zinc batteries.

## Graphical abstract

A hydrophobic surface coating enables the in‐situ formation of a zinc fluoride‐rich solid electrolyte interphase (SEI) in zinc–bromine batteries. This SEI suppresses dendrite formation and water‐induced side reactions, leading to uniform zinc deposition, reduced voltage hysteresis, and extended cycle life. The strategy offers a scalable pathway to high‐efficiency, long‐lasting aqueous zinc battery systems for renewable energy storage.

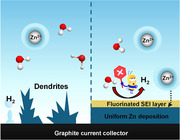

## 1 Introduction

Aqueous batteries are promising candidates for stationary energy storage, offering inherent safety, low cost, and environmental sustainability compared to lithium‐ion batteries, which rely on organic electrolytes. Among the former, zinc–bromine batteries (ZBBs) stand out due to their high energy density, cost‐effectiveness and enhanced safety.^[^ ^1^, ^2^, ^3^, ^4^ ^]^ Additionally, they offer a unique advantage in their zinc‐free anode design. Unlike other zinc battery systems that rely on excessive zinc foils or plates functioning as both anodes and current collectors, ZBBs rely on zinc plating and stripping directly on a current collector during operation.^[^ ^5^ ^]^ This design minimizes the risks associated with zinc metal anodes, such as mechanical fracture, cycling‐induced failure, high negative‐to‐positive ratios, and reduced energy densities. Nevertheless, ZBBs still face challenges, including zinc dendrite formation and water decomposition, which lead to short circuits, electrode degradation, and limited cycle life. Maintaining a healthy Zn plating/stripping process is critical for ensuring long‐term electrochemical performance, directly affecting cycling stability and efficiency.^[^ ^6^ ^]^ The uncontrollable growth of Zn dendrites may lead to: 1) inefficient zinc plating/stripping with the inactive Zn contributing to capacity fade; 2) a high risk of membrane piercing; and 3) internal short circuits, causing self‐discharge and battery failure. Additionally, side reactions such as the hydrogen evolution reaction (HER), associated with the absence of a protective and stable Zn‐ion conductive solid–electrolyte interphase (SEI), further degrade the system during cycling.^[^ ^7^, ^8^, ^9^, ^10^, ^11^, ^12^ ^]^ Addressing these challenges is essential for unlocking the full potential of ZBBs in large‐scale energy storage applications.

In ZBBs, carbon‐based materials are often used as current collectors and are preferred over metallic zinc owing to their cost‐effectiveness, chemical stability, sustainability and versatility (i.e., via doping or functionalization). However, they are subject to degradation in aqueous electrolytes, especially in aggressive chemical environments, leading to reduced capacity, efficiency and limited cycle life, negatively impacting their function and durability.^[^ ^13^ ^]^ Several studies have improved carbon current collectors via surface functionalization to enhance Zn^0^ plating efficiency under electrochemical conditions such as those in ZBBs. Some efforts have been devoted to improving the performance of the negative carbon current collectors via surface activation to increase the electrochemical area accessible to the ZBB electrolyte.^[^ ^6^, ^14^ ^]^ In other studies, poorly conductive carbon current collectors have been coated with highly conductive activated carbon powders to increase the surface area available for electrochemically active redox reactions in ZBBs.^[^ ^15^ ^]^ It is important to note that the SEI, which regulates zinc plating/stripping performance by controlling Zn nucleation and suppressing dendrite propagation and the HER, is a critical factor in preventing battery degradation. However, limited attention has been given to the construction of a stable SEI on carbon current collectors for ZBBs.^[^ ^16^ ^]^

Fluorine‐containing materials have garnered significant attention in battery research due to their unique chemical and physical properties, which contribute to enhanced electrochemical performance and long‐term stability. Fluorine forms one of the strongest covalent bonds with carbon (C─F bond), imparting exceptional chemical stability and degradation resistance. Additionally, its high electronegativity and strong ion–dipole interactions facilitate the formation of robust SEIs, which are crucial to stabilizing electrode surfaces and suppressing undesirable side reactions.^[^ ^10^, ^17^ ^]^ Among fluorinated polymers, perfluoropolyether (PFPE) stands out due to its fully fluorinated ether backbone, ultralow surface energy, and high chemical and oxidative stability.^[^ ^18^ ^]^ Commonly used as a hydrophobic lubricant coating, PFPE is stable in both polar and nonpolar media, with its physical properties tunable through variations in molecular weight, chain architecture, and end groups.^[^ ^19^ ^]^ Compared to other fluorinated polymers such as polyvinylidene fluoride (PVDF) and polytetrafluoroethylene (PTFE), PFPE offers a unique combination of oxidative stability, low surface energy, and chain flexibility. Importantly, under electrochemical conditions, PFPE may undergo partial defluorination, contributing to the in situ formation of a metal fluoride layer on the metal anodes. This inorganic fluoride interphase can mitigate parasitic reactions and improve interface stability, offering functional benefits beyond those achievable with chemically inert PTFE.^[^ ^20^, ^21^ ^]^

In this study, we present a simple yet effective strategy for in‐situ construction of a fluorinated SEI by functionalizing the graphite surface with a hydrophobic PFPE interlayer. The PFPE coating facilitates the formation of a ZnF_2_‐rich SEI, which stabilizes the Zn/electrolyte interface while regulating Zn^2^⁺ transport. The hydrophobic nature of PFPE effectively repels water molecules, thereby mitigating parasitic reactions such as proton reduction. Furthermore, strong electrostatic interactions between Zn^2+^ and F^−^ within the interphase promote robust ionic coordination, reinforcing interfacial stability. As a result, uniform Zn plating/stripping is achieved, supporting stable cycling at a high current density of 25 mA cm^−2^ with an energy efficiency exceeding 79%, highlighting its competitiveness for advanced ZBBs. Furthermore, voltage hysteresis significantly reduces from 285 to 60 mV over up to 1000 cycles with PFPE‐functionalized graphite (PFPE‐G) current collectors. The localized ZnF_2_ formation, combined with the perfluorinated polymer, creates a hybrid interphase on the graphite electrode, representing an innovative approach for the development of advanced anode‐free zinc battery systems.

## 2 Results and Discussion

### 2.1 Validation of Perfluoropolyether‐Functionalized Graphite Performance

Figure S1a (Supporting Information) describes the synthesis steps for the PFPE‐G electrode. The bare graphite (Bare‐G) was initially treated via oxygen plasma to activate the surface energy and introduce several oxygen‐containing functional groups (i.e., carboxyl and hydroxyl [−OH]) to remove adsorbed gases and contaminants that could weaken adhesion (Figure S1b, Supporting Information). The oxygen plasma also etched and roughened the carbon surface, introducing structural defects and enhancing wettability.^[^ ^22^ ^]^ The oxygen plasma treatment of the graphite surface enhanced its chemical affinity and enabled stable attachment of the hydroxyl‐terminated PFPE. The PFPE coating is chemically inert and does not alter the bulk pH of the ZnBr_2_ electrolyte. However, it effectively isolates the electrode surface from direct water contact, which helps mitigate local proton reduction and suppresses HER, despite the weakly acidic nature of the electrolyte.

**Figure 1:**
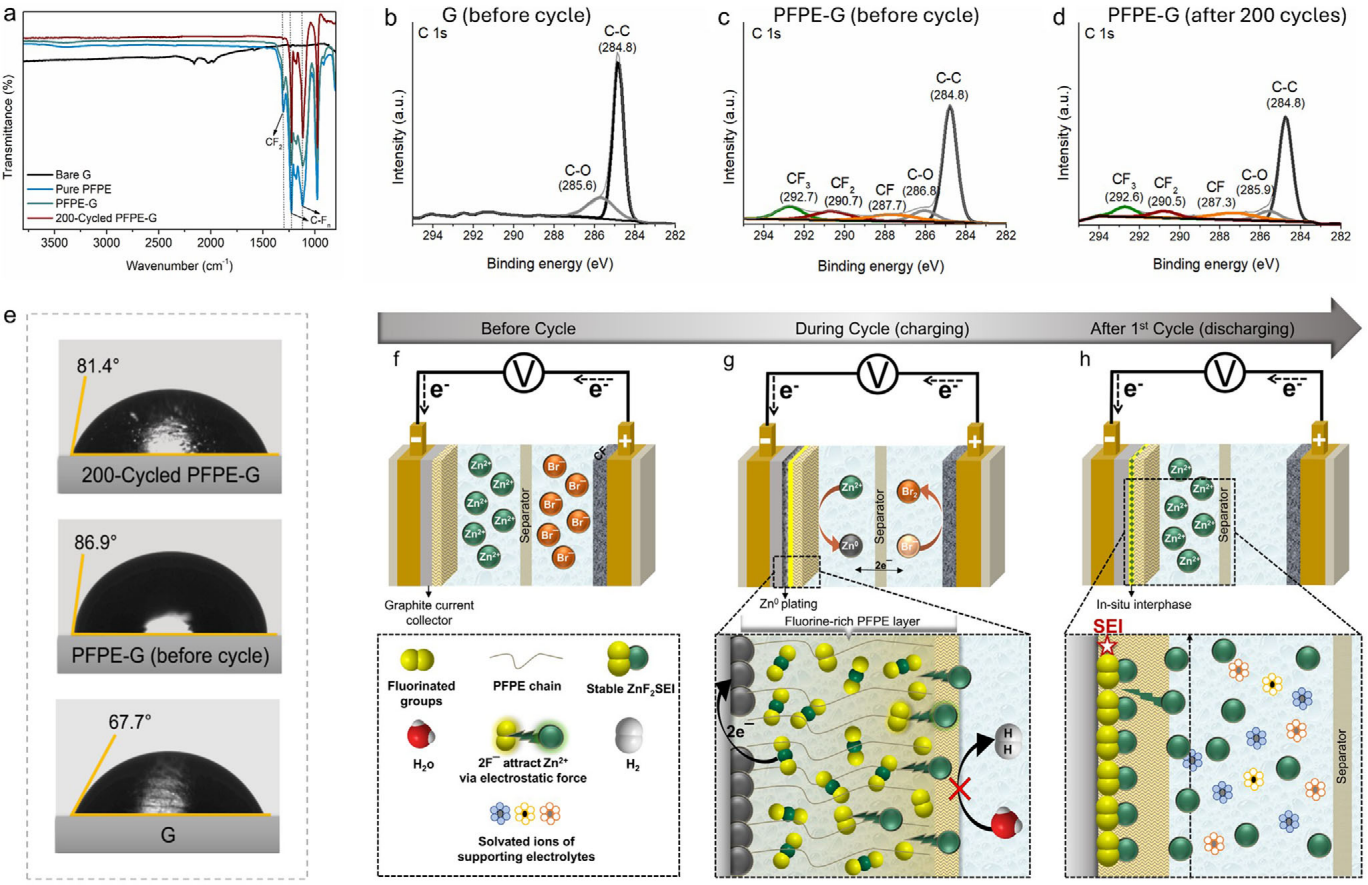
Chemical characterization of the perfluoropolyether‐functionalized graphite (PFPE‐G) surface compared with that of bare graphite, analyzed by a) attenuated total reflection–Fourier transform infrared spectroscopy of the G, PFPE and PFPE‐G surfaces before cycling and after 200 cycles. b–d) X‐ray photoelectron spectra of C1s for the G and PFPE‐G current collectors before and after 200 cycles. In (c), the PFPE‐G electrode was left at room temperature for several weeks to investigate the coating stability and durability. Physical properties (wettability) of PFPE‐G. e) Contact angle measurements when a 5 µL droplet of 2.5 m ZnBr_2_ electrolyte solution was dispersed onto the G and the PFPE‐G surfaces before and after 200 cycles. A schematic illustration of a proposed mechanism of PFPE coating at different cycle stages. f) The fluorinating agent (PFPE) layer is applied before the cycle. g) The Zn^2+^ ion‐transporting pathways through the PFPE layer before the reduction process to metallic Zn^0^ during the charging phase, with an inset description. h) In‐situ fluorinated interphase formed after the first cycle (terminated at the discharged state), with an inset description.

To confirm the successful functionalization of PFPE on graphite collectors, multiple analysis techniques were employed to examine the surface composition and chemical interactions. Attenuated total reflectance–Fourier‐transform infrared spectroscopy (FTIR; **Figure** **1**a) provides direct evidence of chemical modifications upon PFPE deposition. The adsorption of ─OH stretching was observed in the region of 3200–3600 cm^−1^. The weak ─OH band associated with OH‐terminated PFPE was significantly reduced after functionalization, primarily due to the thinner coated layer compared to that in the bulk phase, which lowers the detectable ─OH concentration in FTIR. Additionally, surface interactions may induce molecular rearrangement, affecting signal intensity without entirely depleting the ─OH groups. The adsorption of C─F_n_ (*n* = 2,3) bending shows as sharp peaks in Figure 1a, where F‐components can later interact with the graphite surface via van der Waals forces, and contribute to stabilizing the adsorbed species on the graphite surface.^[^ ^23^ ^]^ Peaks indicative of PFPE were observed in the PFPE‐G and remained present after many repeated cycles (i.e., 200). The peak measurements of 1230 and 1116 cm^−1^ correspond to the stretching vibrations of the trifluoromethyl (CF_3_) group,^[^ ^24^, ^25^ ^]^ and their presence after long‐term cycles indicated a strong attachment between the coating PFPE materials and the graphite substrate. A difluoromethylene (CF_2_) stretching vibration at 1300 cm^−1^ was observed before cycling and exhibited a partial decrease in intensity after discharge, indicating consumption of CF_2_ groups associated with ZnF_2_ formation. This finding was further confirmed by X‐ray Photoelectron Spectroscopy, where distinct peaks corresponding to C‐F_n_ (*n* = 1, 2, and 3) configurations were observed in the C 1s spectrum after PFPE deposition (Figure 1b,c). The emergence of these fluorinated signals indicates the successful formation of a chemically robust PFPE interfacial layer on the graphite surface. Notably, after 200 cycles, the C 1s spectrum (Figure 1d) continued to exhibit C─F_n_ features, albeit with a slight shift toward lower binding energies and a modest decrease in peak intensity. This observation suggests partial surface evolution but confirms the overall retention of fluorinated functionalities, highlighting the long‐term chemical durability and interfacial stability of the PFPE coating under extended battery cycling conditions.

To assess the physical characteristics of the PFPE coating, the surface wettability, thickness, and morphology of Bare G and PFPE‐G were evaluated. Contact angle measurements using the sessile drop method with a 2.5 m ZnBr_2_ electrolyte revealed an initial contact angle of 67.7° for the Bare graphite, which increased to 86.9° after PFPE functionalization. After extended cycles, PFPE‐G maintained a higher contact angle (81.4°), indicating the durability of the hydrophobic coating (Figure 1e), compared to the Bare G (Figure S2, Supporting Information). Confocal microscopy (Figure S3, Supporting Information) provided insights into the coating thickness, observing a measured height of ≈2 µm, representing the average thickness of the PFPE layer on the graphite surface.

Cross‐sectional scanning electron microscopy (SEM) images of PFPE‐G samples, left for eight weeks at room temperature, followed by additional drying in a vacuum oven overnight, further validated the strong adhesion of PFPE and its effectiveness in smoothing the typically rough graphite surface (Figures S4 and S5, Supporting Information). Despite graphite surfaces possessing advantageous features for ZBBs compared to other materials, irregularities and the microscopically inhomogeneous surface resulting from the manufacturing process often promote nonuniform zinc plating, which can trigger localized accumulation and dendrite formation. However, by applying a PFPE layer, this work introduces multi‐functional aspects to boost performance. In addition to enhancing stability and efficiency in Zn plating and HER suppression, the PFPE's protective and functional properties improve interfacial compatibility, promote selective ion transport and minimize side reactions. This method also aims to create a smooth and uniform graphite surface with an even potential distribution, enabling a more uniform zinc plating/stripping process to help minimize zinc accumulation and dendrite formation, and ultimately enhance ZBBs’ performance and longevity. Density functional theory (DFT) calculated the adsorption energy of PFPE on graphite (Figure S6a, Supporting Information) and graphite with an oxygen defect (Figure S6b, Supporting Information), further supporting spectroscopic and morphological results. The negative value (−0.549 eV) indicates the interaction between PFPE and graphite with an oxygen defect, revealing the spontaneous adsorption process of PFPE.

### 2.2 In‐Situ Solid Electrolyte Interphase Formation and Ion Transport Mechanism

An efficient SEI layer is crucial to achieving good cycling stability, longevity, high performance, and battery safety.^[^ ^26^ ^]^ However, understanding these characteristics in aqueous electrolytes (i.e., ZnBr_2_) requires further investigation, especially when modifications are introduced to either the electrode surface or the electrolyte. The HER remains a major challenge in ZBB developments, as it degrades both the electrolyte and the electrode (Figure S7, Supporting Information), and reduces the overall efficiency. The excessive generation of hydrogen gas bubbles depletes Zn^0^ nucleation sites, obstructing electrode‐electrolyte contact. Since the HER requires H_2_O adsorption before proton reduction, minimizing water adsorption is key to suppressing HER activity. The introduction of the PFPE layer enhances surface hydrophobicity while facilitating Zn^2+^ incorporation with fluorine to form a protective ZnF_2_ interphase. The strong electronegativity of fluorine in PFPE aids in the desolvation of the Zn(H_2_O)_6_^2+^ at the electrode surface, facilitating the diffusion of the Lewis acidic Zn^2+^, while minimizing its exposure to water molecules.^[^ ^27^, ^28^ ^]^ Figure 1f,h illustrates the proposed reaction mechanism of PFPE interaction with the graphite surface. The fluorinated groups (CF_2_, CF_3_) in PFPE create electrostatic attractions for Zn^2+^ ions, guiding them toward the electrode surface for controlled deposition.

**Figure 2:**
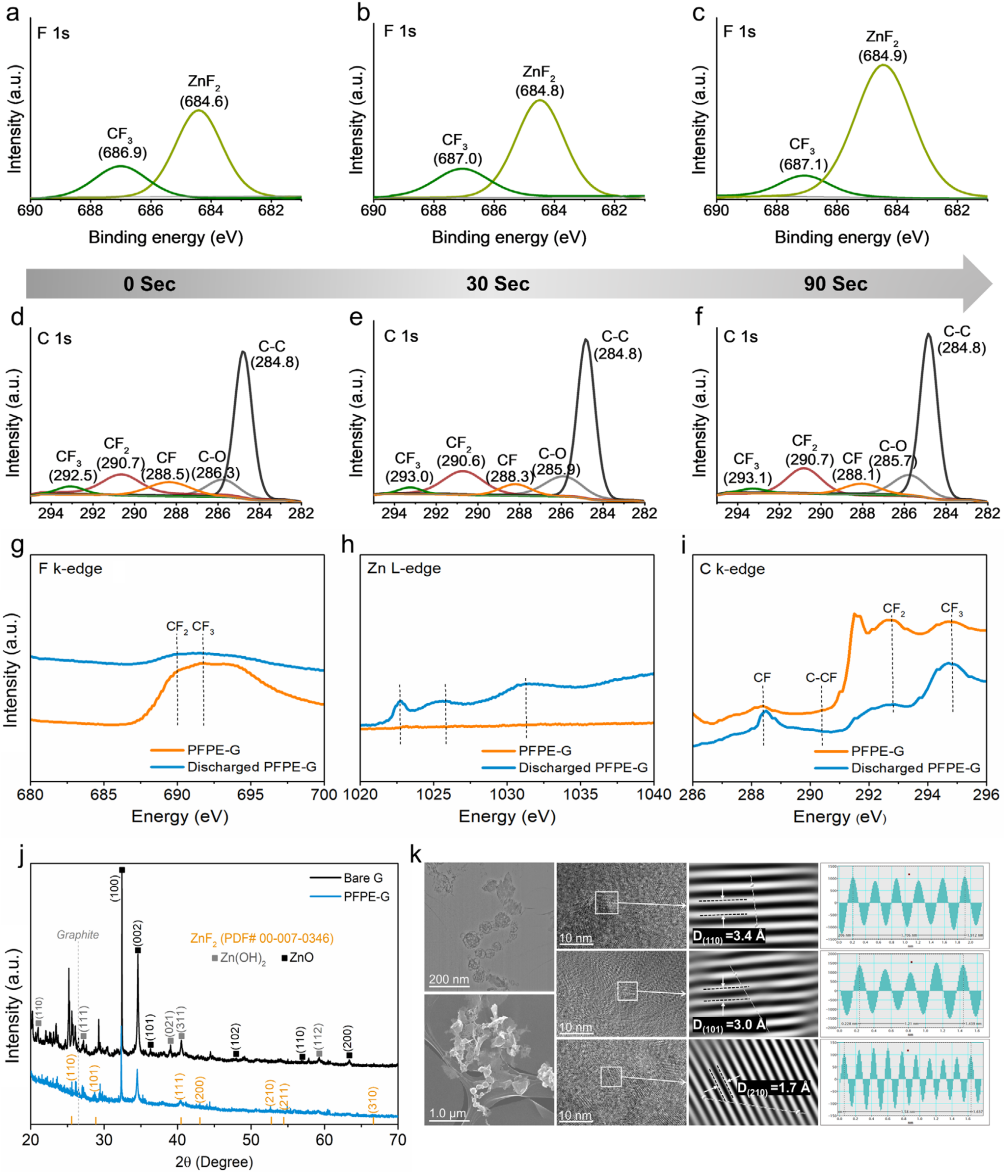
Characterization of the ZnF_2_ interphase. a–f) X‐ray photoelectron spectroscopy (XPS) depth profiles of (a–c) F 1s and (d–f) C 1s after 0, 30, and 90 s of Ar^+^ sputtering for perfluoropolyether‐coated graphite (PFPE–G) samples after initial cycling, where Zn^0^ was stripped. g–i) Synchrotron soft X‐ray absorption spectrum of fluorine K‐edge, zinc L‐edge and carbon K‐edge for PFPE‐G current collectors before and after discharge. j) XRD patterns of discharged bare G and PFPE‐G current collectors. k) TEM and high‐resolution TEM images of the discharged PFPE‐G current collector, showing lattice fringes with corresponding d‐spacings for different ZnF_2_ planes.

In this study, F‐rich SEI formation was confirmed through X‐ray photoelectron spectroscopy depth profiling, as illustrated in **Figure** **2**a,f. In the initial scan of the discharged PFPE‐G surface (Figure 2a,d), ZnF_2_ significantly increased while CF_3_ groups diminished with increasing depth, suggesting that the bonding environment changed, potentially due to the formation of ZnF_2_ replacing carbon in CF_2_ within the SEI (Figure 2c); this result is in good agreement with the literature.^[^ ^29^ ^]^ This result indicates that this conversion is dynamically favorable owing to the stability of ZnF_2_ under the study's electrochemical conditions, providing enhanced protection over long‐term cycles. Due to the dynamic Zn plating/stripping process and the surface roughness of the graphite substrate, it is difficult to precisely measure or define the SEI thickness. The interphase likely exhibits a compositional gradient, with a ZnF_2_‐rich inorganic layer near the metal surface and more organic or polymeric components toward the electrolyte, contributing to both ion transport and side reaction suppression.

To further confirm the interphase formed on the cycled (1^st^ discharged) PFPE‐G current collector after Zn stripping, synchrotron soft x‐ray absorption spectroscopy was used to analyze the oxidation states and electronic structure of F, Zn, C, and O (Figure 2g,i). The fluorine K‐edge spectrum (Figure 2g) exhibited two pronounced absorption features at ≈690.1 and 691.7 eV, which assigned to the 1s →*σ* * transition in CF_2_ and CF_3_ moieties, respectively.^[^ ^30^ ^]^ After discharge, these peaks decreased in intensity, suggesting that the fluorinated species underwent chemical transformation. This change may have been attributed to interactions between fluorine atoms from CF_2_ and CF_3_ groups and zinc at the interphase during cycling, forming new bonding configurations, including ZnF_2_. In the zinc L‐edge region, characteristic peaks at 1,022.7 and 1,025 eV confirmed the presence of Zn^2+^ species in the SEI (Figure 2h).^[^ ^31^, ^32^, ^33^ ^]^ In the carbon K‐edge spectrum (Figure 2i), four distinct resonances were observed at 288.4, 291.1, 292.7, and 294.8 eV, which can be assigned to *σ* excitations associated with C─F, C─C, CF_2_, and CF_3_ groups, respectively.^[^ ^30^ ^]^ Peaks at 293.3 and 293.8 eV before and after discharge indicated variations in the carbon coordination environments. After discharge, the CF_2_ peak exhibited a pronounced decrease, indicative of the consumption of internal CF_2_ units, likely through conversion into ZnF_2_ within the SEI. Conversely, the CF and CF_3_ peaks showed modest increases, suggesting fragmentation or structural reorganization of residual fluorinated moieties, enriching terminal CF and CF_3_ functionalities. This structural change remained stable over hundreds of cycles, confirming the durability of the coating layer (Figure S8, Supporting Information).

X‐ray diffraction (XRD) patterns of the discharged electrodes (Figure 2j) reveal pronounced structural distinctions between the bare G and PFPE‐G current collectors. The PFPE‐G electrode exhibited sharp reflections indexed to the (110), (101), and (220) planes of tetragonal Rutile‐type ZnF_2_ (PDF# 00‐007‐0346), confirming the formation of crystalline ZnF_2_ as a dominant SEI component on the PFPE‐modified surface. In contrast, the bare G electrode displayed additional diffraction features attributable to ZnO and Zn(OH)_2_ (JCPDS card No. 38–0385), indicative of parasitic by‐product formation, in agreement with previously reported observations.^[^ ^34^ ^]^ These species were markedly suppressed in the PFPE‐G sample, underscoring the protective role of the ZnF_2_‐rich SEI in mitigating undesirable Zn corrosion pathways, likely by passivating reactive sites and limiting contact between metallic Zn and trace water. Transmission electron microscopy (TEM) and high‐resolution TEM of the discharged PFPE‐G electrode (Figure 2k) further corroborated the presence of crystalline ZnF_2_, revealing lattice fringes with d‐spacings of 3.4, 3.0, and 1.7 Å corresponding to the (110), (101), and (210) planes, respectively.

A clear difference in the crystalline orientations with an average size of ≈200 nm and abundant crystalline directions was also observed in the TEM image for the bare G and PFPE‐G (Figure S9, Supporting Information). As illustrated in Figure S10 and Table S2 (Supporting Information), SEM–energy‐dispersive X‐ray spectroscopy further confirmed the elemental composition of the interphase. The atomic ratio of fluorine (6.4%) was estimated to be compatible with that of zinc (3.9%), matching the expected stoichiometry of these elements in the interphase. Although the above analyses confirm the presence of ZnF_2_ and other SEI components after electrochemical conditioning, they reflect the interphase composition at a fixed point in time. Elucidating the dynamic formation mechanism during initial cycles remains challenging due to the complexity of the Zn interface and the sensitivity of fluorinated species. Future in situ studies will be essential to resolve the temporal evolution of the SEI and clarify its formation pathways.

### 2.3 Evaluation of Zinc‐Plating Morphology and Hydrogen Evolution Behavior

**Figure 3:**
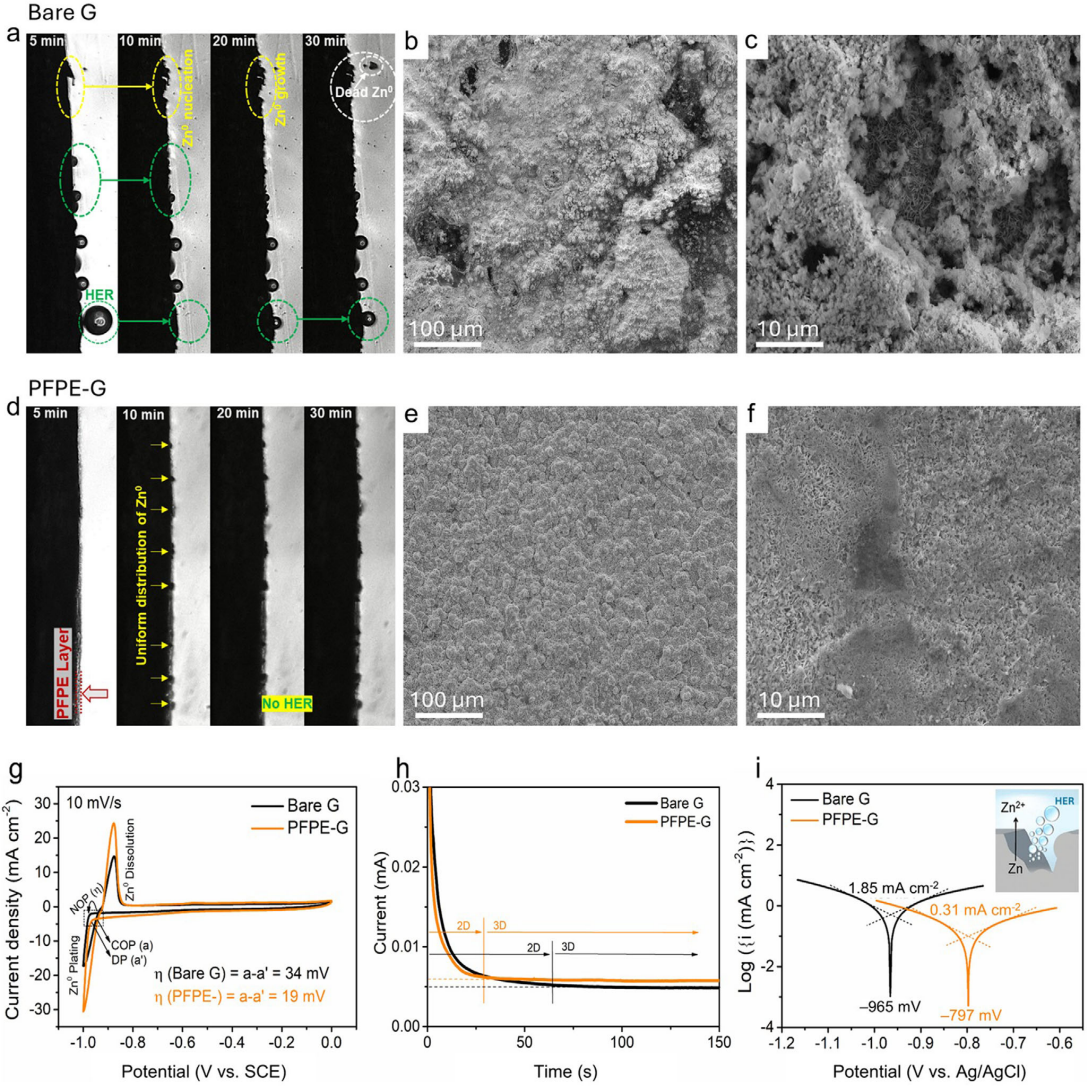
Visualization of the hydrogen evolution reaction and zinc plating morphology for the bare graphite (G) and perfluoropolyether‐coated G (PFPE‐G). a,d) Operando in‐situ observation of varying charging durations for Bare‐G and PFPE‐G electrodes in symmetrical cells, each charged at 5 mA for 30 min, captured using an optical microscope. b,e) Low‐magnification (100×) and c,f) high‐magnification (1000×). Scanning electron microscopy illustrates the zinc plating morphology on the Bare‐G and PFPE–G current collectors in a full nonflow ZBB. g) Cyclic voltammetry curves of the Zn half‐cell on the Bare‐G and PFPE‐G current collectors, recorded versus a saturated hydrogen calomel reference electrode, highlighting differences in redox behavior. h) Chronoamperometry analysis of the Bare‐G and PFPE‐G (treated) current collectors in a three‐electrode system at a fixed potential of −150 mV. i) Simulated Tafel analysis obtained from linear sweep voltammetry for the Bare‐G and PFPE‐G (Figure S13, Supporting Information) in a three‐electrode configuration, using Ag/AgCl as the reference electrode.

Uniform Zn plating and stripping on the current collector are crucial to enhancing Zn^2+^ utilization, maximizing the discharge capacity and increasing the cycling stability of ZBBs.^[^ ^35^ ^]^ To investigate zinc‐plating behavior and the associated HER side reaction, operando optical microscopy was performed (**Figure** **3**a,d; Figure S11, Supporting Information). The Bare‐G and PFPE‐G current collectors were charged at a current density of 5 mA cm^‒2^ for 30 min. On the bare G surface, gas bubbles appeared within the first 5 min of charging, increasing in number throughout the plating process. These bubbles occupied Zn^0^ nucleation sites, leading to uneven zinc deposition with vertically oriented, sharp‐ended structures (Figure 3a). In contrast, the PFPE layer altered the zinc‐plating morphology, eliminating hydrogen bubbles and dendritic zinc growth. Operando observation confirmed uniform Zn plating on the PFPE‐G over a 30‐min period (Figure 3d). The Zn‐plating microstructure and surface topography were further examined using SEM. Low‐ and high‐magnification SEM imaging revealed that Zn deposition on the Bare‐G current collector exhibited a highly porous and uneven morphology, with pore formation likely driven by hydrogen evolution and gas escape during electroplating (Figure 3b,e; Figure S12, Supporting Information). In contrast, the Zn plating on the PFPE‐G produced a significantly more compact, uniform and fine‐grained structure (Figure 3c,f). Together, the optical and SEM analyses highlight the vital role of the fluorinated PFPE‐G interfacial layer in modulating Zn nucleation and growth, enabling uniform plating morphology and reinforcing interfacial stability in ZBB systems.

Cyclic voltammetry (CV) of zinc half‐cells was conducted to examine Zn^2+^ electroplating behaviour on Bare‐G and PFPE‐G current collectors (Figure 3g). A three‐terminal glass cell was used, with either Bare‐G or PFPE‐G (1 cm^2^) as the working electrode, platinum foil as the counter electrode, and a saturated calomel electrode (SCE) as the reference. The electrolyte solutions were purged with nitrogen gas before testing to remove dissolved oxygen.^[^ ^36^ ^]^ Zinc plating involves a series of surface reactions, including nucleation and growth, where nucleation overpotentials cause the deposition potential of zinc to be more negative than the standard potential of Zn^2+^|Zn^0^.^[^ ^23^ ^]^ The CV results (Figure 3g) revealed key differences between the Bare‐G and PFPE‐G. First, Zn^0^ electroplating on the PFPE‐G initiated at 930 mV, suggesting a lower onset potential. In contrast, Zn^0^ electroplating on the Bare‐G commenced at 950 mV, indicating a higher kinetic barrier. Second, the nucleation overpotential (η), defined as the potential difference between points a and a′, was significantly lower for PFPE‐G (19 mV), compared with Bare‐G (34 mV). This finding suggests that the energy barrier required to initiate Zn^0^ nucleation is lower on the PFPE‐G current collector, which promotes smaller, more uniform nuclei, leading to a smoother and denser Zn^0^ plating layer with suppressed dendrite formation.^[^ ^37^ ^]^ Although ZnF_2_ is generally considered an insulating material, in the SEI, it likely forms a thin, nanostructured layer with defects or grain boundaries that allow Zn^2+^ transport. Its incorporation within a composite interphase, including polymeric and organic components, helps suppress parasitic reactions while maintaining plating kinetics. The observed lower onset potential on PFPE‐G suggests that the SEI formed is both stable and permissive to Zn nucleation and growth.

The chronoamperometry curves (Figure 3h) provide insights into the nucleation, diffusion, and growth behaviour of Zn^2+^ ions and adsorbed Zn atoms on the electrode surface.^[^ ^37^ ^]^ The Bare‐G current collector, the current remained relatively stable for ≈65 s before gradually decaying. This behavior suggests a prolonged two‐dimensional (2D) Zn^2^⁺ diffusion pathway, leading to ion accumulation and the formation of dendrites due to the localized electric field enhancement, commonly referred to as the “tip effect.”^[^ ^38^ ^]^ In contrast, the PFPE‐G could stabilize the current in a shorter time (≈25 s), demonstrating that the random diffusion of Zn^2+^ ions was rapidly suppressed. The stabilization of the current further indicated a transition from 2D surface diffusion to a quasi‐3D diffusion process, where Zn deposition was well regulated, with a more controlled Zn growth mechanism.

Linear sweep voltammetry (LSV) measurements (Figure S13, Supporting Information) revealed that the PFPE‐G electrode exhibits a more negative onset potential for the HER compared to bare graphite. Tafel extrapolation (Figure 3i), derived from the LSV response, revealed a positive shift in the corrosion potential (E_corr_​) from ‐965 to ‐797 mV following the introduction of the PFPE layer. Additionally, the corrosion current density (I_corr​_) decreased by 1.54 mA cm^−2^ (from 1.85 to 0.31 mA cm^−2^), indicating improved anticorrosion performance.^[^ ^38^, ^39^ ^]^ The calculated corrosion rate for the PFPE‐G current collector was 4.77 mm per year (mmpy), compared to the much higher rate of 27.82 mmpy for the Bare‐G, highlighting substantial material degradation in the absence of protection. This improvement was further supported by visual evidence of degradation in the cycled Bare‐G over 500 cycles (Figure S13a, Supporting Information, inset).

### 2.4 Electrochemical Evaluation of Nonflow ZBB Performance

**Figure 4:**
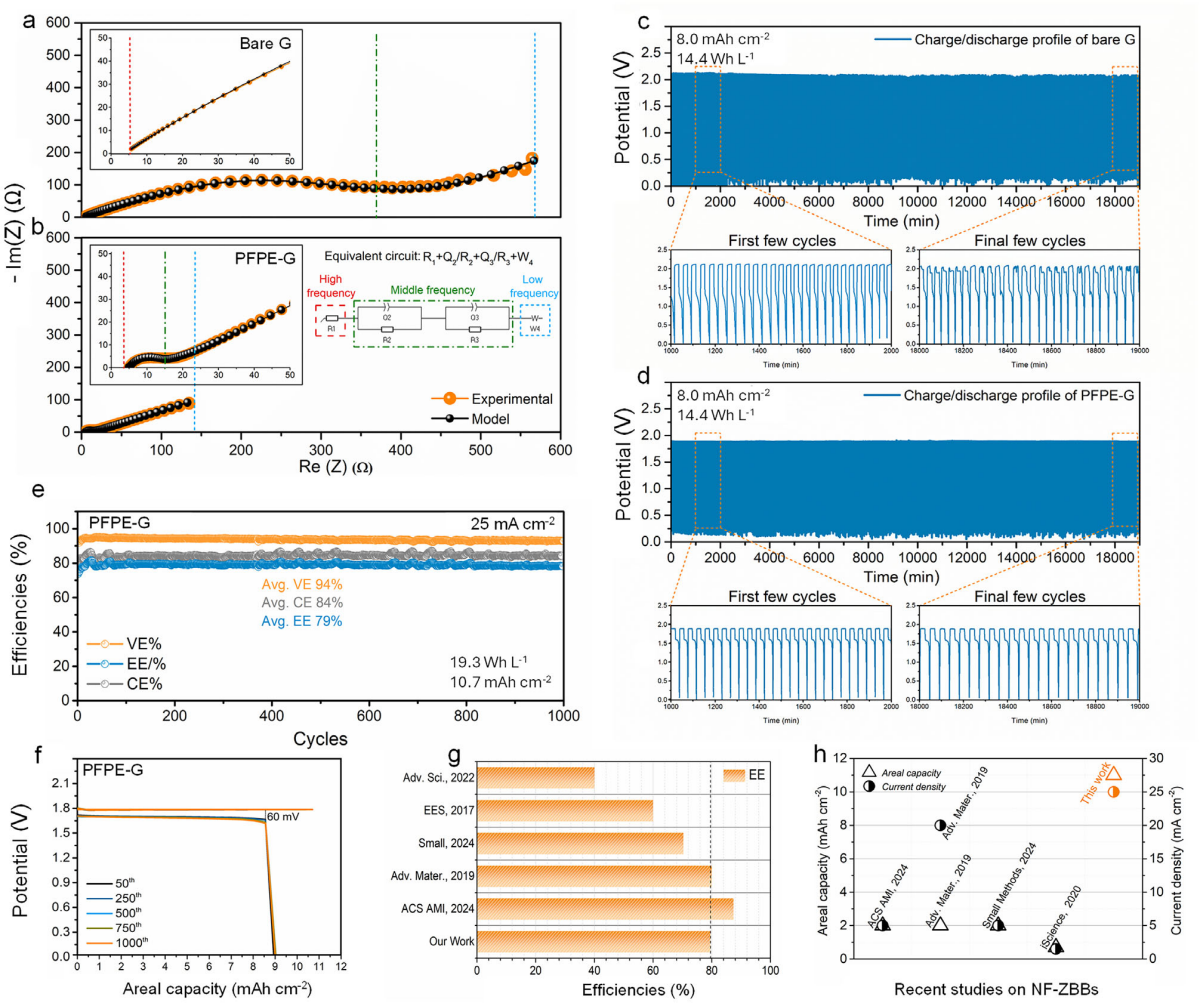
a,b) The electrochemical impedance spectroscopy (EIS) of (a) Bare‐G and (b) PFPE‐G), performed in a nonflow ZBB (NF‐ZBB) system and measured at 100 kHz–10 mHz by applying an AC potential (10 mV) between a working and reference electrode, with the simulated equivalent circuit to fit the EIS signals. c,d) Cycling performance of (c) bare‐G and (d) PFPE‐G in NF‐ZBBs at a current density of 25 mA cm^−2^ and an areal capacity of 8.0 mAh cm^−2^, under 1 m electrolyte concentrations. e) Coulombic, energy and voltage efficiencies and f) voltage profile of PFPE‐G over different cycles at 25 mA cm^−2^ and an areal capacity of 10.7 mAh cm^−2^, under 0.5 m electrolyte concentrations. g,h) Performance comparisons between the metrics of this study's battery and those of previously reported ZBBs at various efficiencies, current densities and areal capacities.

To further assess the electrochemical performance of PFPE‐G in ZBBs, full cells were assembled with both bare‐G and PFPE‐G electrodes and performed electrochemical impedance spectroscopy (EIS) after 10 consecutive cycles. Measuring EIS at this stage provides insight into stabilized device resistance, encompassing contributions from both electrodes, evolving SEI, and interfacial effects under realistic operating conditions. Nyquist plots, which typically display one to three semicircles corresponding to distinct time constants in different frequency regions,^[^ ^40^, ^41^ ^]^ were analyzed using a simulated equivalent circuit. Table S3 (Supporting Information) presents the resistance values for the Bare‐G and PFPE‐G. In these results, the diameter of the semicircle at middle frequencies significantly decreased in the PFPE‐G cell (**Figure** **4**a,b; Table S3, Supporting Information), indicating that the fluorinated coating layer forms a hydrophobic, corrosion‐resistant barrier. This layer suppresses parasitic reactions and prevents excessive SEI growth, thereby facilitating faster ion transfer at the interface and reducing polarization. Additionally, the low frequency resistance, associated with diffusion process of Zn^2+^ ions, was substantially reduced, suggesting improved ion transport and interfacial stability. This enhancement is particularly important to maintaining long‐term cycling performance. A slight decrease in the R_1_ from 1.1 to 0.9 Ω for Bare‐G and PFPE‐G, respectively, was also observed, confirming that the bulk electrolyte and cell components' resistance remained largely unchanged. Overall, these results demonstrate that the PFPE‐G layer significantly improved the electrochemical performance by facilitating charge transportation, promoting faster Zn^2+^ kinetics and reducing interfacial resistance.

The long‐term cycling performance of ZBBs was evaluated for G|CF and PFPE‐G|CF configurations with two different electrolyte concentrations (0.5 and 1 m) to enable a comparative analysis of PFPE under different electrolyte conditions. As displayed in Figure 4c, the Bare‐G‐based cell initially exhibited stable cycling but then demonstrated capacity fluctuations and decay over extended cycling, primarily due to severe HER and dendrite formation, which led to current collector degradation (>500 cycles, Figure S14a, Supporting Information, inset). In contrast, the PFPE‐G based ZBB demonstrated stable cycling performance for over 300 h (500 cycles) (Figure 4d) at an areal capacity of 8.0 mAh cm^−2^ and a current density of 25 mA cm^−2^. It achieved an average Coulombic efficiency of 94%, with a lower voltage hysteresis of 200 mV (Figures S14b and S15, Supporting Information), which was a significant reduction compared to the 600 mV hysteresis observed in the Bare‐G system, highlighting the improved electrochemical stability and efficiency of the PFPE‐G current collector. The reduced voltage hysteresis not only improves energy efficiency by lowering irreversible energy losses but may also mitigate battery heat generation during operation. Furthermore, while round‐trip time largely depends on current and capacity, the enhanced reaction kinetics associated with reduced hysteresis could support faster cycling under practical conditions.

To explore performance under more favorable ion transport conditions, a lower‐concentration 0.5 m ZnBr_2_ electrolyte was employed. At a higher areal capacity of 10.7 mAh cm^−2^ and a current density of 25 mA cm^−2^, the PFPE‐G cell exhibited an average Coulombic, voltage and energy efficiencies of 84%, 94%, and 79%, respectively (Figure 4e). On the other hand, a higher CE of 94% with 76% EE was achieved under the same PFPE‐G configuration with 1 M ZnBr_2_ over 500 cycles (Figure S14b, Supporting Information), outperforming the performance reported in high‐impact studies (Table S4, Supporting Information). In addition, the maximum stored energy per unit volume (energy density) reached ≈20 Wh L^−1^, demonstrating stability for over 1000 cycles. A significantly lower voltage hysteresis of 60 mV was further observed (Figure 4f) in the PFPE‐G system compared to the 285 mV in the Bare‐G, which also exhibited reduced capacity retention over 1000 cycles (Figure S16, Supporting Information). This improvement is attributable to the use of a less concentrated 0.5 m ZnBr_2_ electrolyte, which minimizes ionic crowding and enhances mass transport under dynamic conditions. Additionally, the PFPE‐G coating facilitated selective Zn^2+^ transport while suppressing the HER, further contributing to higher efficiency. In contrast, when using a 1 m ZnBr_2_ electrolyte at an areal capacity of 8.0 mAh cm^−2^ and the same current density of 25 mA cm^−2^, the increased ionic interactions and water activity led to a larger potential hysteresis between the charge and discharge processes (Figure S14a, Supporting Information), negatively impacting cycling efficiency. While higher concentrations of ZnBr_2_ (e.g., 1 m) can theoretically improve energy density, the 0.5 m electrolyte exhibited longer cycling stability, likely due to reduced voltage hysteresis and improved electrode/electrolyte compatibility, underscoring a trade‐off between energy density and long‐term performance.

Figure 4g,h benchmarks this study's ZBB performance against that of state‐of‐the‐art non‐flow ZBBs across key electrochemical metrics, including EE, areal capacity, and current densities.^[^ ^42^, ^43^, ^44^, ^45^, ^46^ ^]^ These charts underscore the superiority of PFPE‐G in achieving high‐performance and durable ZBBs, demonstrating this technology's potential for next‐generation energy storage applications. Performance comparisons between the metrics of this study's battery and those of previously reported ZBBs at various efficiencies, current densities, areal capacities, cell configuration, electrolyte composition, and electrode materials are presented in Table S4 (Supporting Information). Beyond nonflow ZBBs, the efficacy of PFPE‐G was further validated in a flow‐type ZBB (Figure S17, Supporting Information), which demonstrated stable cycling performance at 5 mA cm^−2^ with improved Zn^0^ plating morphology. Comparative analysis of flow‐type ZBBs using Bare‐G as the current collector confirms the advantages of PFPE‐G in enhancing plating uniformity (Figures S18–S20, Supporting Information).

## 3 Conclusion

In summary, this study demonstrated a multifunctional perfluorinated polymer coating for graphite current collectors, significantly enhancing the performance of anode‐free ZBBs. This modification facilitates Zn^2+^ ion transport via electrostatic attraction with the fluorine regions, creating an in‐situ ZnF_2_ interphase layer. Additionally, the polymer acts as a protective layer, suppressing undesirable side reactions and promoting long‐term cycling stability. As a result, the zinc plating morphology remains uniform, with minimal dendrite growth. Electrochemical analysis confirms substantial improvements, including a significantly reduced voltage hysteresis of 60 mV at an areal capacity of 10.7 mAh cm^−2^, and exceptional cycling stability over 1000 cycles. Furthermore, a high EE of 79% is achieved at a current density of 25 mA cm^−2^, outperforming the most recent state‐of‐the‐art static ZBBs. These findings underscore the effectiveness of this research work's approach in enhancing ZBB performance across various configurations, with broader potential for improving interfacial control in hybrid flow batteries and modular zinc‐based systems. This study provides new insights into carbon‐based current collector design and may accelerate the industrial development of ZBBs for next‐generation anode‐free zinc battery applications.

## Conflict of Interest

The authors declare no conflict of interest.

## Supporting information



Supporting Information

## Data Availability

The data that support the findings of this study are available from the corresponding author upon reasonable request.
