# Supplementary material for: High‐Performance Zinc–Bromine Rechargeable Batteries Enabled by In‐Situ Formed Solid Electrolyte Interphase
Source: Adv Sci (Weinh). 2025 Sep 29;12(46):e08646. doi: 10.1002/advs.202508646 (PMC12697910; doi:10.1002/advs.202508646)
Supplement: Supplementary file 1 — Supporting Information [file ADVS-12-e08646-s001.pdf]

Supporting Information

High-performance Zinc-Bromine Rechargeable Batteries enabled by in-situ formed solid electrolyte interphase

Norah S. Alghamdi, Xiyue Peng, Xingchen Yang, Shengyong Gao, Yongxin Huang, Shuangbin Zhang, Tong'en Lin, Cheng Zhang, Ian R. Gentle, Lianzhou Wang, Bin Luo*

N. S. Alghamdi, X. Peng, X. Yang, S. Gao, Y. Huang, S. Zhang, T. Lin, C. Zhang, L. Wang, B. Luo

Australian Institute for Bioengineering and Nanotechnology, The University of Queensland, Brisbane QLD 4072, Australia. E-mail: b.luo1@uq.edu.au

L. Wang, B. Luo

School of Chemical Engineering, The University of Queensland, Brisbane QLD 4072, Australia.

N. S. Alghamdi, I. R. Gentle

School of Chemistry and Molecular Biosciences, The University of Queensland, Brisbane QLD 4072, Australia.

N. S. Alghamdi

Department of Chemistry, Faculty of Science, Imam Mohammad Ibn Saud Islamic University (IMSIU), Riyadh 11564, Saudi Arabia.

L. Wang

Department of Applied Biology and Chemical Technology, Faculty of Science, The Hong Kong Polytechnic University, Hong Kong SAR, P.R. China

Experimental Section

Preparation of electrolytes

The initial positive and negative electrolytes ranged from 0.5 to 1 mol L^{-1} $ZnBr_2$, 0.2 mol L^{-1} $ZnCl_2$, 0.4 mol L^{-1} KCl and 0.4 mol L^{-1} 1-methyl-1-ethylpyrrolidinium bromide in deionised water. $ZnBr_2$ served as the primary source of Zn^{2+} and Br^{-} ions. KCl and $ZnCl_2$ were added as supporting salts to enhance electrolyte conductivity, while the quaternary ammonium compound acted as a complexing agent to stabilise free bromine in the solution during the charging process.

Preparation of Perfluoropolyether-Coated Graphite Current Collectors and Battery Assembly

The Graphite (G) foil and carbon felt (CF) were cut into 0.2 cm^2 pieces for use as negative and positive current collectors, respectively, in two different sizes of Swagelok-type cells. Surface functionalisation of the G electrode with a perfluoropolyether (PFPE) layer was adapted from previous studies with slight modifications[1]. Briefly, PFPE (PFPE AL-2, Mw = 2000) was preheated at 60 °C for 30 min to reduce viscosity, followed by drop-casting approximately 2 μL cm^{-2} of PFPE oil onto the G surface (Figure S1). Coating thickness control was verified using confocal microscopy (Figure S3), which revealed a measured height of approximately 2 μm, representing the average thickness of the PFPE layer on the graphite surface. The functionalised current collectors were immediately ready for use in various battery configurations.

The MS Glass Fiber membrane (0.7 μm, 70 mm) was used as the separator. This study primarily focused on non-flow zinc–bromine batteries (NF-ZBBs) assembled in Swagelok-type cells using an electrolyte volume of 0.2 mL, and an areal capacity of up to ~11 mAh cm^{-2} and a maximum current density of 25 mA cm^{-2}. Table S1 presents the cell performance parameters for the Swagelok-type NF-ZBB cells used here. The parameters included the electrode radii (r), electrolyte concentrations, state of charge, electrolyte volume, electrode area, current density, capacity, areal capacity and energy density. All current collectors and membranes were soaked in the electrolyte before use. Cycling performance and stability assessments were performed for the ZBBs featuring G|CF and PFPE-G|CF configurations at a high current density of 25 mA cm^{-2} and different areal capacities. Galvanostatic charge/discharge tests were conducted using a LAND battery test system (CT2001A, Wuhan, China) at 25°C.

Additionally, a number of tests were conducted using zinc-bromine flow batteries (ZBFB) configurations to validate the applicability of the approach across different ZBB setups. The ZBFBs were also tested using G|CF or PFPE-G|CF as the negative and positive electrodes, respectively, in a C-flow cell configuration (1 cm^2). Each electrolyte (20 mL) was circulated at a flow rate of 20 mL min^{-1}. After disassembly, the current collectors were thoroughly rinsed with deionised water to remove residual salts and prepared for further investigations.

Material characterisation

Attenuated total reflectance–Fourier-transform infrared spectroscopy (ATR-FTIR) was conducted using a Cary 630 FTIR instrument (Agilent Technologies) equipped with an attenuated total reflectance sampling accessory. This system collects IR spectra of materials in the range of 400–4000cm^{-1} and was used to identify the functional groups and chemical bonds present in the samples. Spectra were collected in the range of 3900–900 cm^{-1} at a resolution of 4 cm^{-1}, with 32 scans averaged per sample. Contact angle measurements were performed using a DataPhysics Instruments OCA 14EC/B goniometer. A 5 µL droplet of 2.5 M $ZnBr_2$ aqueous solution was dispensed onto bare graphite (G) and PFPE-functionalised graphite (PFPE-G) surfaces at 25 °C. The contact angle was measured immediately after droplet deposition using the SCA202 software with the Eclipse fitting method for profile analysis. The thickness of the PFPE coating on graphite substrates was evaluated using confocal laser scanning microscopy (LEXT OLS4000, Olympus). Surface conductivity measurements of bare G and PFPE-G were performed via four-point probe analysis. For *in-situ*/*operando* optical microscopy analysis, a quartz optical window cell (diameter: 21 mm, Model: TOB-19004) was used to visualise the impact of hydrogen gas bubbles on zinc plating morphology. In this symmetric configuration (G|G and PFPE-G|PFPE-G), the electrodes were fully immersed in 2 M $ZnBr_2$ aqueous electrolyte. The zinc plating behaviour and surface evolution were monitored using a Nikon Eclipse Ti-U inverted microscope while applying a constant current of 5 mA cm^{-2} for 30 minutes with a LAND battery test system. The analysis primarily focused on HER bubble formation.

X-ray photoelectron spectroscopy (XPS) analysis was conducted using a Kratos Axis Ultra spectrometer equipped with a monochromatic Al Kα excitation source to investigate the chemical states and elemental compositions of discharged bare G and PFPE-G electrodes. Depth profiling of the solid–electrolyte interphase (SEI) layer was performed using Ar^+ sputtering ($Ar1000^+$) under the following conditions: 10 keV beam energy, 30 seconds per etching cycle, and a 2 mm raster size. All electrodes were stored in a vacuum oven (≥ 8 hours)

prior to characterisation to eliminate moisture. X-ray diffraction (XRD) measurements were carried out using a Bruker D8 Advance MKII diffractometer with Cu Kα radiation (λ = 1.5405 Å) at a scan rate of 2° min^{-1} to determine the crystalline phases of discharged bare G and PFPE-G electrodes.

High-resolution transmission electron microscopy (HRTEM) was employed to visualise the microstructure and crystallinity of discharged bare G and PFPE-G electrodes. Samples were dispersed in ethanol by sonication before imaging. Energy-dispersive X-ray spectroscopy (EDS) elemental analysis and mapping were performed using a Hitachi SU3500 scanning electron microscope equipped with an Oxford X-Max SDD detector to examine the spatial distribution of key elements on the electrode surfaces. This helped identify variations in composition between bare graphite and PFPE-G after electrochemical cycling.

Atomic force microscopy (AFM) measurements were performed in air using a Bruker Dimension XR atomic force microscope equipped with a Nanoscope™ 3D ADC5 Multimode system (Veeco Instruments Inc.), operating in tapping mode. A PFTUNA conductive probe with a sharp tip (tip curvature radius <10 nm) was employed. Topography images were flattened, and surface features were analysed using the section analysis tool in Nanoscope Analysis software. This technique was used to evaluate surface roughness and morphological differences between Zn deposits on bare G and PFPE-G surfaces following electrochemical testing.

Synchrotron-based soft X-ray absorption spectroscopy (XAS) was carried out at the Australian Synchrotron (ANSTO) to investigate the fluorine K-edge, zinc L-edge, carbon K-edge, and oxygen K-edge of ex situ PFPE-G electrode samples before cycling and after discharge. These measurements were performed at the Soft X-ray Beamline using total electron yield (TEY) mode. Spectra were processed using the measured photon energy, the sample drain current, and reference foil calibrations to ensure data accuracy.

Electrochemical Tests

Electrochemical impedance spectroscopy (EIS) was carried out over a frequency range of 100 kHz to 10 mHz using a three-electrode setup. Bare graphite or PFPE-G (1 cm^2) served as the working electrode, with a platinum foil and a saturated calomel electrode (SCE) as the counter and reference electrodes, respectively. Prior to testing, all solutions were purged with nitrogen and maintained under an inert atmosphere to remove dissolved oxygen. Cyclic voltammetry (CV) was performed in the potential range of 0 to -1.5 V vs. SCE at a scan rate of 10 mV/s to evaluate the redox activity of Zn^{2+}/Zn^0 in both electrode types.

Chronoamperometry (CA) was carried out at a fixed potential of -150 mV vs. Ag/AgCl to investigate Zn^{2+} ion nucleation, diffusion, and hydrogen evolution reaction (HER) behaviour. Linear sweep voltammetry (LSV) measurements were performed using bare G or PFPE-G as the working electrode, platinum foil as the counter electrode, and Ag/AgCl as the reference electrode. A scan rate of 10 mV s^{-1} was applied. The resulting data were used to construct Tafel plots for evaluating corrosion behaviour.[2]

The corrosion rate (CR) was determined using the corrosion current density (I_{corr}) obtained from Tafel extrapolation, following Faraday's law. The calculation was conducted using Biologic software under the corrosion and Tafel analysis module, employing the standard corrosion rate equation. A conversion factor ($K=3.27\times10^{-3}$) was applied to express the corrosion rate in mm/year (mmpy).

The parameters considered in the analysis included:

- Molar mass of Zn (M=65.38g/mol)
- Number of electrons transferred (n=2)
- Faraday's constant (F=96,485)
- Density of Zn ($\rho=7.14$ g/cm^3)
- Equivalent weight (eq. wt.) of Zn, calculated as atomic weight/valency

The corrosion rate was calculated for a 1 cm^2 electrode surface area, providing the final value in mm/year (mmpy).

All electrochemical measurements were conducted using a Bio-Logic SP-300 potentiostat, and data were extracted, processed, and fitted using EC-Lab software (version 11.50).

Energy density calculation

The energy density in watt-hours per litre (Wh L^{-1}) was calculated using this formula:

$$Energy\ density\ (Wh\ L^{-1}) = \frac{Energy\ capacity\ (Wh)}{Volume\ (L)} \qquad (1)$$

The Energy capacity (*Wh*) was determined according to this formula:

$$Wh = V \times Ah \qquad (2)$$

V is the average nominal voltage of the battery, and Ah is the total charge the battery can store in ampere-hours.

Theoretical capacity calculation

The theoretical capacity Q_t (mAh) was calculated using the following formula

$mAh = n \times c \times v \times F/3{,}600 = ncv \times 26.8\ (mAh)$ (3) [3]

n represents the number of electrons transferred in the redox reactions, c represents the electrolyte concentration (M), V is the electrolyte volume used (ml) and F is the Faraday constant (96,485 C mol^{-1}).

Theoretical Calculations

To analyse the adsorption energy, the density functional theory (DFT) calculations have been performed using the Vienna ab initio simulation package (VASP 6.1) and the visualization of VESTA software.[4, 5] The electron exchange-correlation function was treated using a generalized gradient approximation (GGA) in the form proposed by Perdew, Burke, and Ernzerhof (PBE).[6] The energy cutoff of the plane waves was set to 500 eV. All presented geometry optimizations are obtained until the maximum displacement below 0.005 Å, energy tolerance below 1×10^{-5} Ha, and the maximum forces lower than 0.01 eV $Å^{-1}$. The lattice constants larger than 15 Å along the x- and y-directions and vacuum region of about 20 Å along the z-direction was adopted. The k-point grid of 3 × 3 × 1 was selected for geometry optimizations. The adsorption energy (E_{ads}) between substrate and adsorbed molecule was defined by the following formula:

$$E_{ads}=E_{tot}-E_{Vo\text{-}C}-E_{PFPE}$$

Where E_{tot}, $E_{Vo\text{-}C}$, and E_{PFPE} are the total energy of the C containing oxygen vacancies and adsorbed PFPE molecule, C containing oxygen vacancies, and PFPE molecule, respectively.

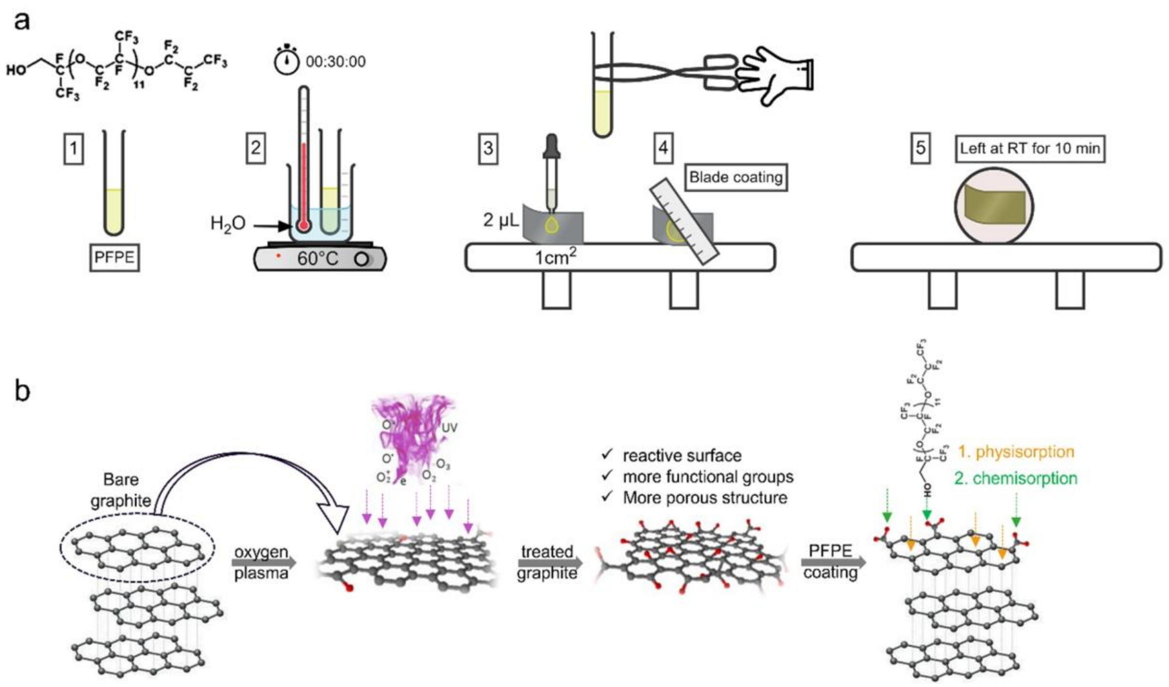

Figure S1. (a) Experimental steps describing the perfluoropolyether (PFPE)-functionalised graphite (G) current collector (PFPE-G). (b) Treatment of the G electrode with oxygen plasma to introduce oxygen-containing functional groups, enabling PFPE attachment. The OH-terminated group of PFPE is consumed, with PFPE being physiosorbed into surface defects and chemisorbed through functional groups on the graphite surface. Some parts of the image are taken from a previous study.[7]

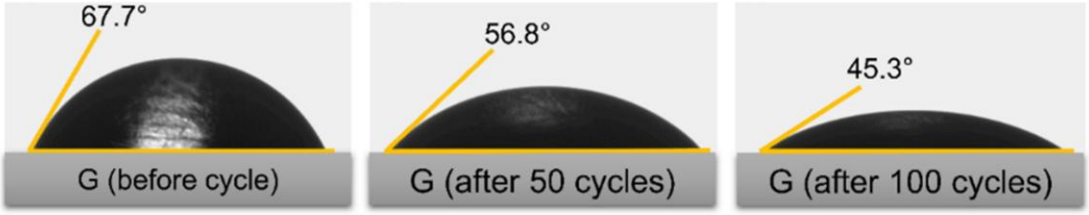

Figure S2. Contact angle measurements with a 5-μL droplet of 2.5 M $ZnBr_2$ electrolyte solution dispersed onto the bare graphite before and after selected cycles. The decrease in contact angle indicates the degradation of the graphite surface over cycling.

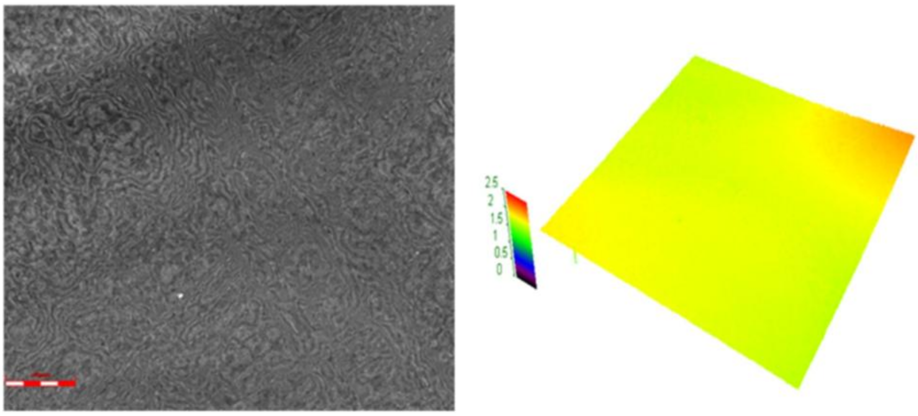

Width [μm]	Height [μm] (Thickness)	Length [μm]	Angle [°]
129.073	1.819	129.085	0.807

Figure S3. Laser scanning microscopy images produced the two-dimensional (2D; left) and 3D (right) perfluoropolyether (PFPE) layers of the PFPE-coated graphite (G) current collector. The measurements were based on a laser scan range of 2.5 μm (in the vertical direction). The average thickness (height) of the cross-sectional area of the PFPE-G is 1.819 μm.

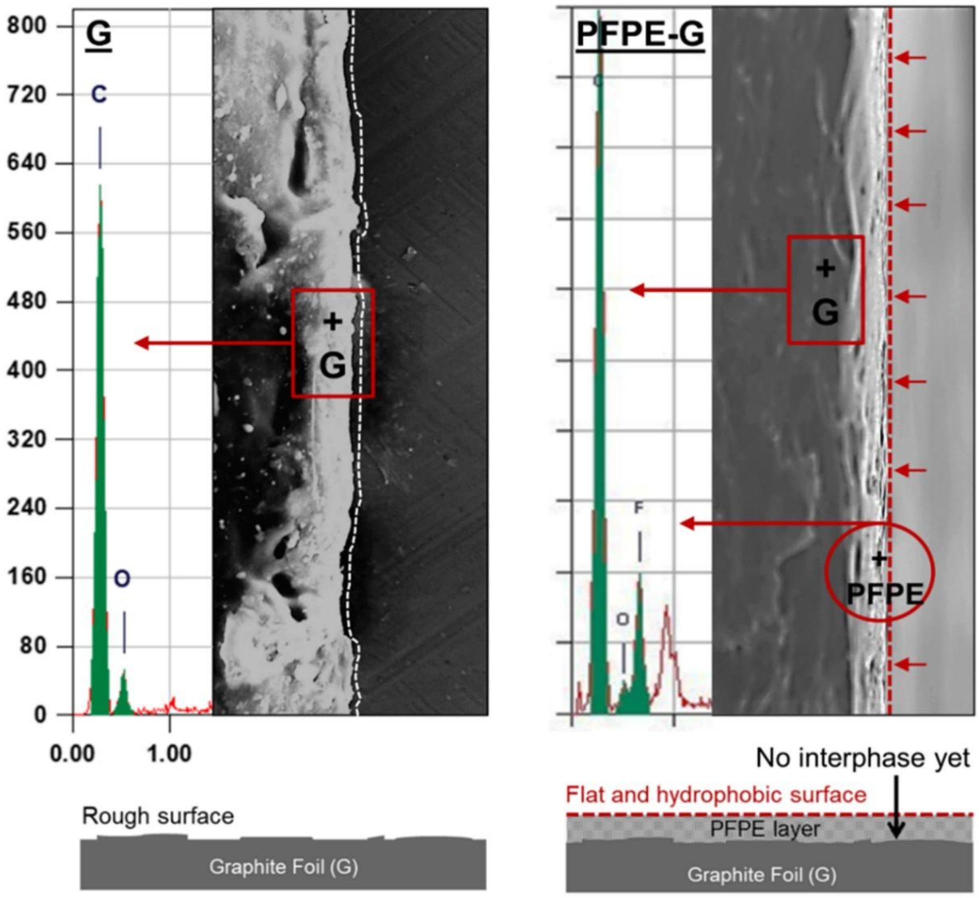

Figure S4. Cross-sectional scanning electron microscopy with the respective energy-dispersive X-ray spectroscopy elemental analysis images and illustrative diagrams of the graphite and perfluoropolyether-coated G surfaces before cycling.

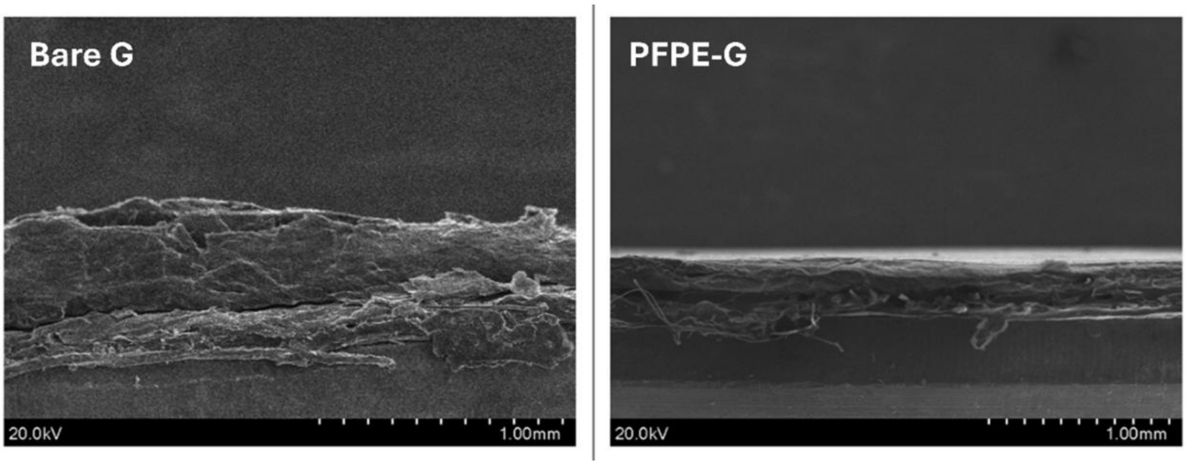

Figure S5. Cross-sectional scanning electron microscopy images of graphite (G) and perfluoropolyether-coated G surfaces after one cycle.

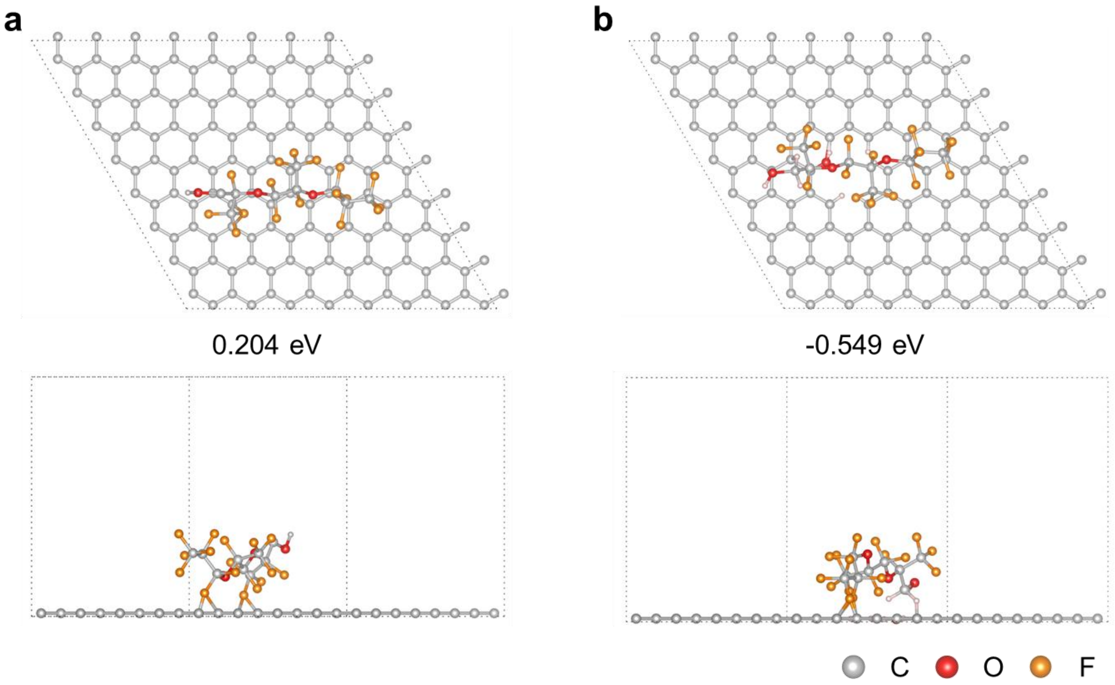

Figure S6. DFT calculated adsorption energy of PFPE monomer on (a) graphite and (b) graphite with oxygen defect.

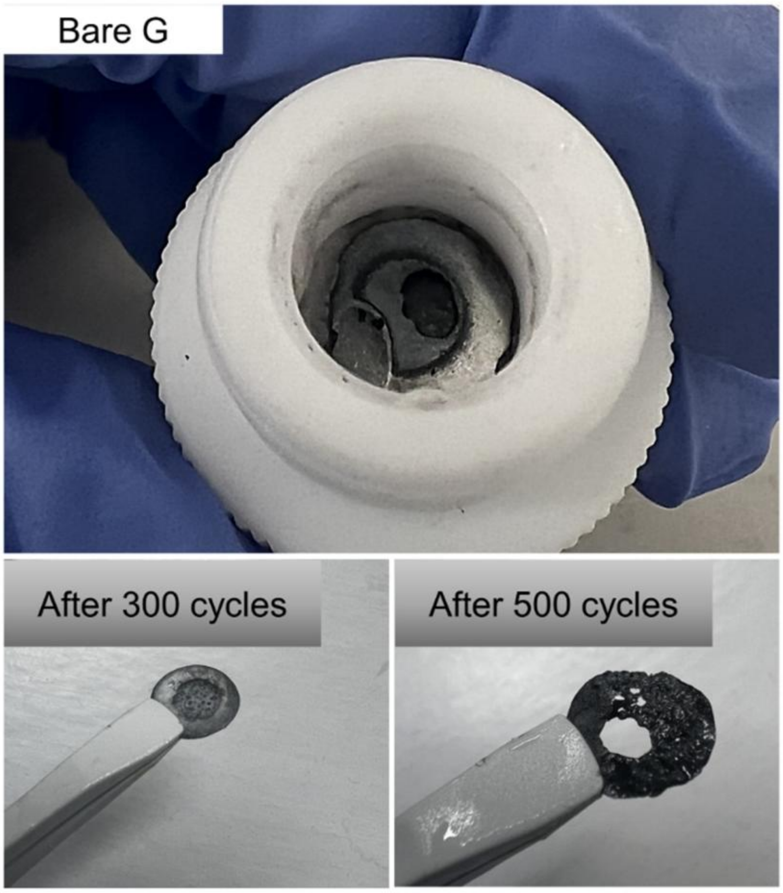

Figure S7. Degraded/damaged graphite-current collector after selected cycles, attributed to the hydrogen evolution reaction (HER), highlighting the need for a protective layer that allows ion transport while functioning as a barrier against the HER.

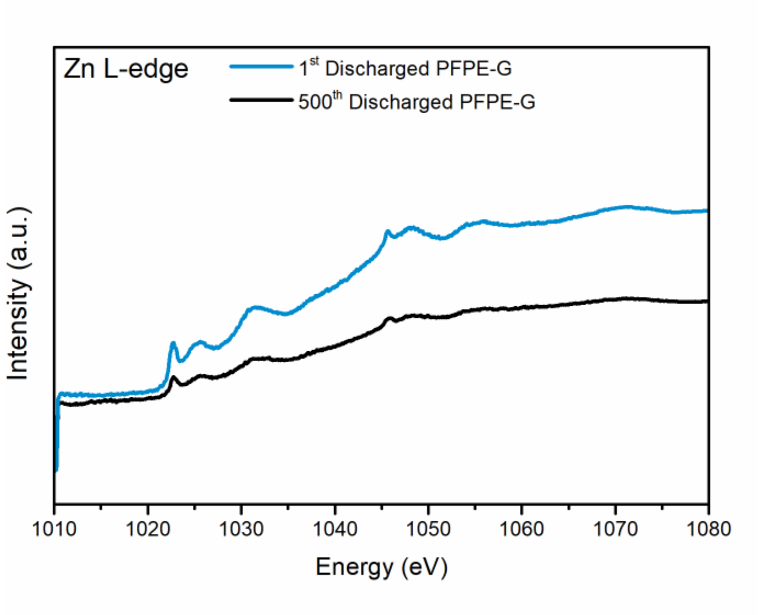

Figure S8. Synchrotron soft x-ray absorption spectra of zinc L-edge for the perfluoropolyether-coated graphite (PFPE-G) after 500 cycles, confirming the stability of the solid electrolyte interphase layers.

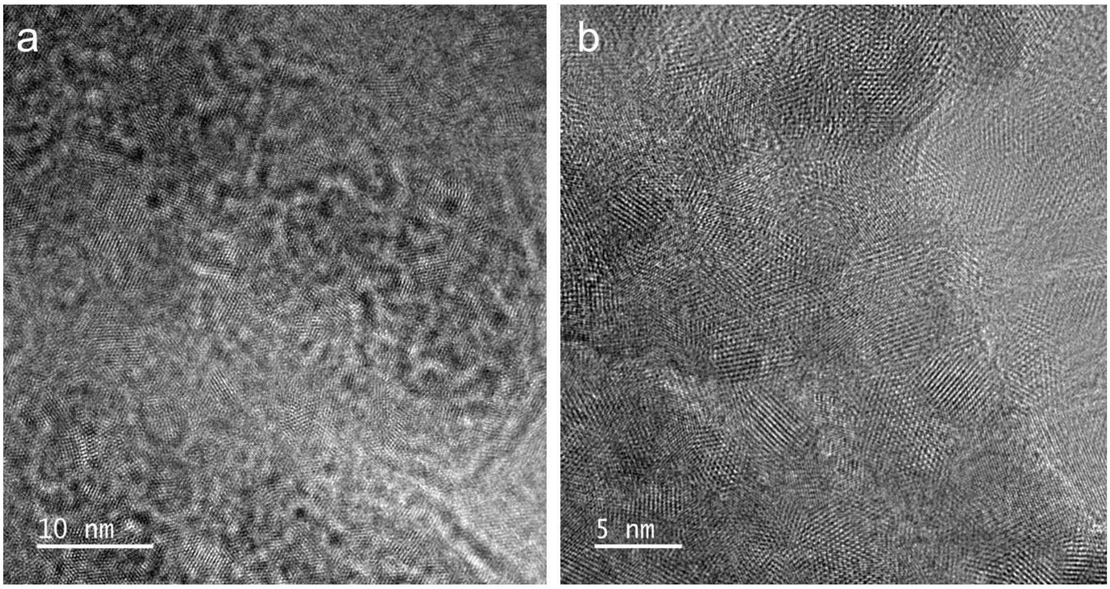

Figure S9. Transmission Electron Microscopy images reveal abundant crystalline directions in the discharged PFPE-G sample.

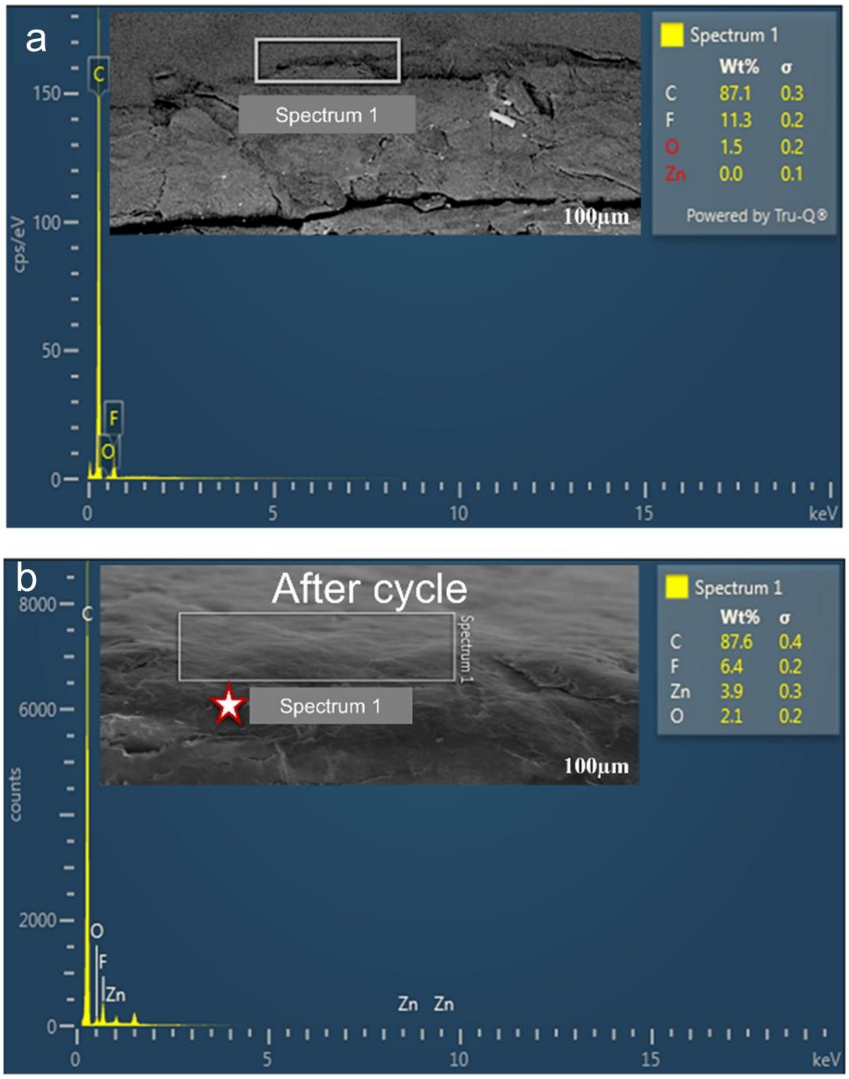

Figure S10. Energy-dispersive X-ray spectroscopy elemental mapping for perfluoropolyether-coated graphite (a) before and (b) after the cycling process.

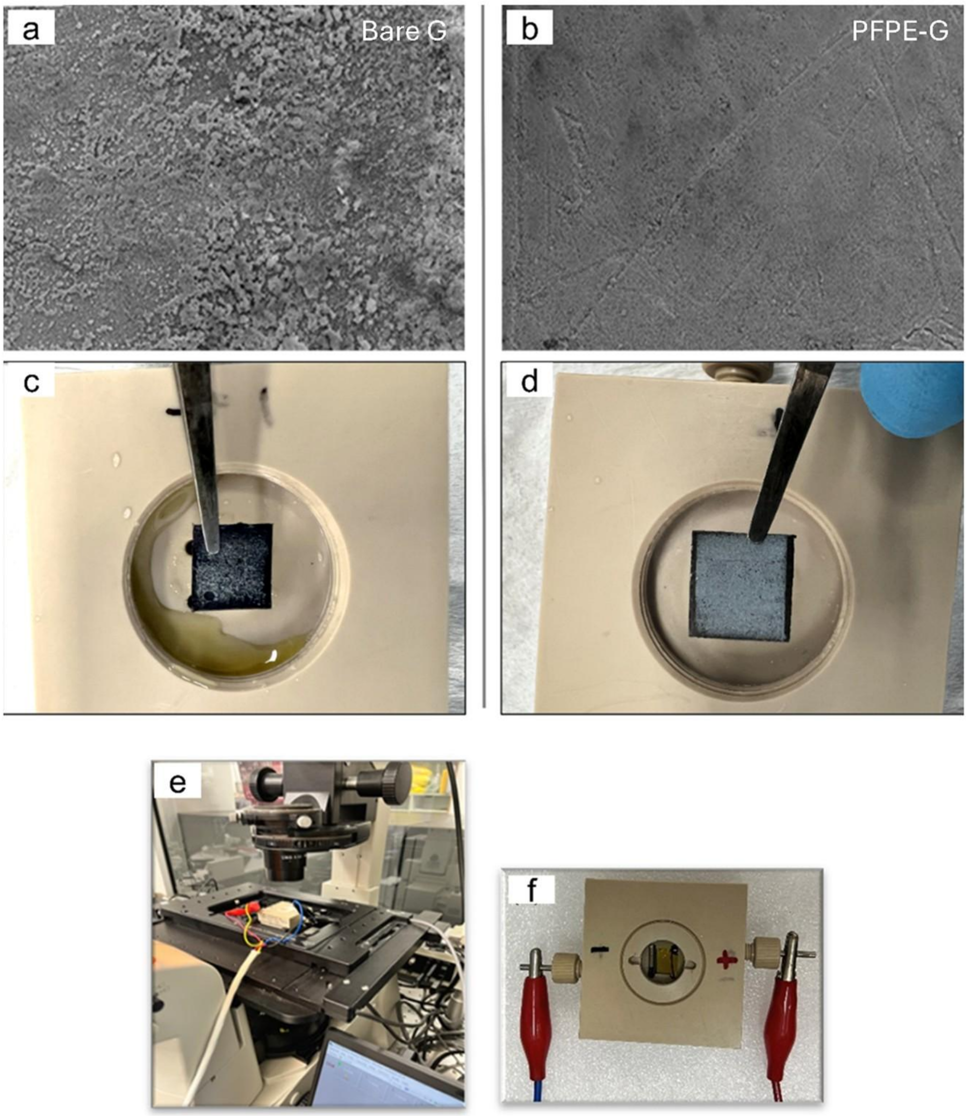

Figure S11. (a, b) Scanning electron microscopy and (c, d) digital images of the bare graphite (G) and perfluoropolyether-coated G (PFPE-G) in symmetrical cells charged at 5 mA for 30 min under in-situ optical microscopy measurement. Digital image of (e) Nikon Eclipse Ti-U inverted microscope connected to (f) A quartz optical window cell with symmetric electrodes fully immersed in 2.5 M $ZnBr_2$ electrolyte.

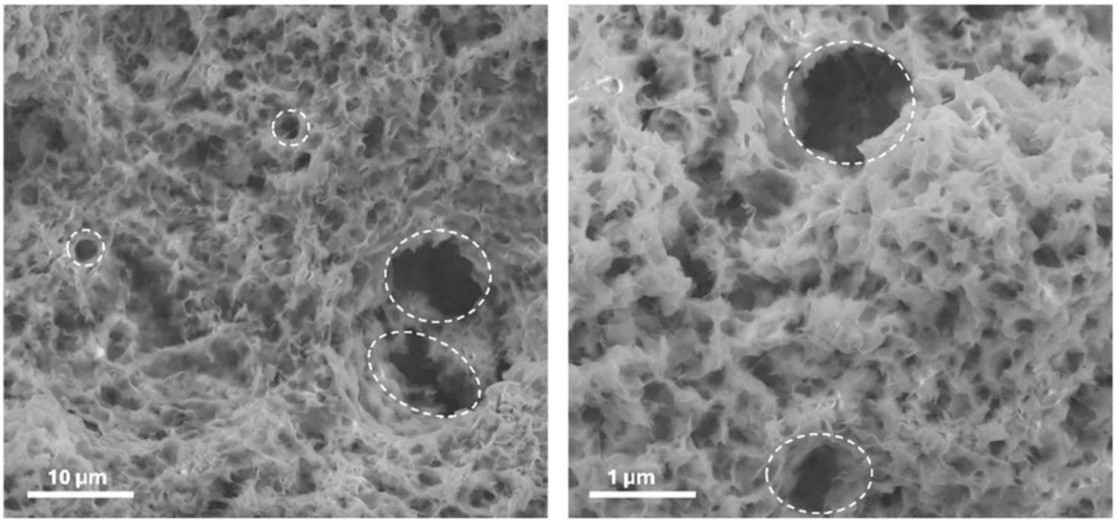

Figure S12. Scanning electron microscopy images of the bare graphite in a full non-flow zinc–bromine battery after extended charging cycles. The images reveal pores of varying diameters within the Zn^0 layer plated on the bare graphite surface, confirming the competition between the hydrogen evolution reaction and Zn^0 plating behaviour.

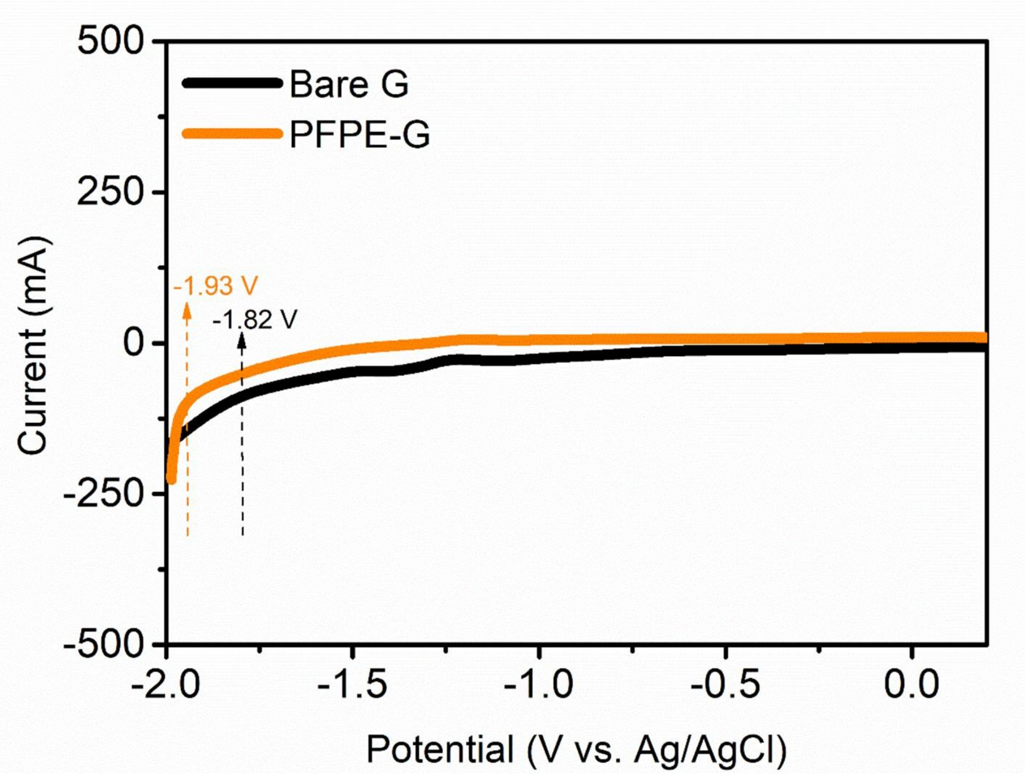

Figure S13. Linear sweep voltammetry for the Bare-G and PFPE-G in a three-electrode configuration, using Ag/AgCl as the reference electrode.

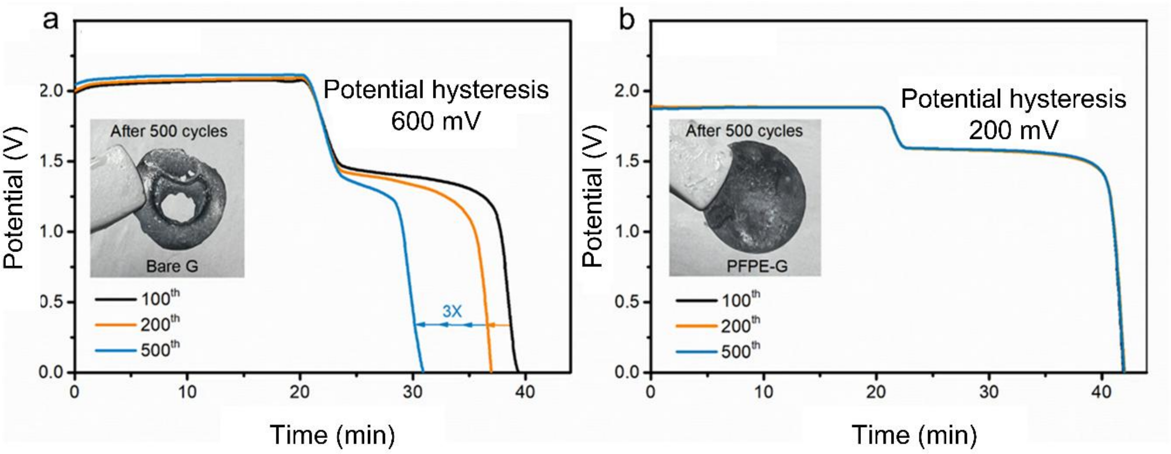

Figure S14. Voltage profiles of (a) graphite and (b) perfluoropolyether-coated graphite at a current density of 25 mA cm^{-2} with 1 M $ZnBr_2$ electrolyte.

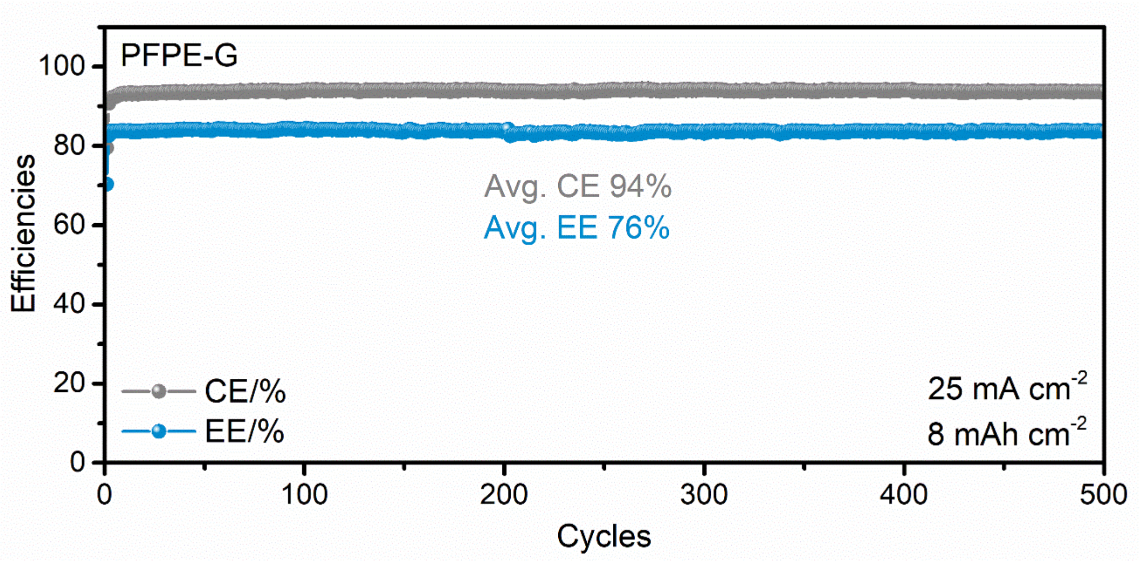

Figure S15. Coulombic and energy of the perfluoropolyether-coated graphite at 25 mA cm^{-2} and an areal capacity of 8 mAh cm^{-2} with 1 M $ZnBr_2$ electrolyte.

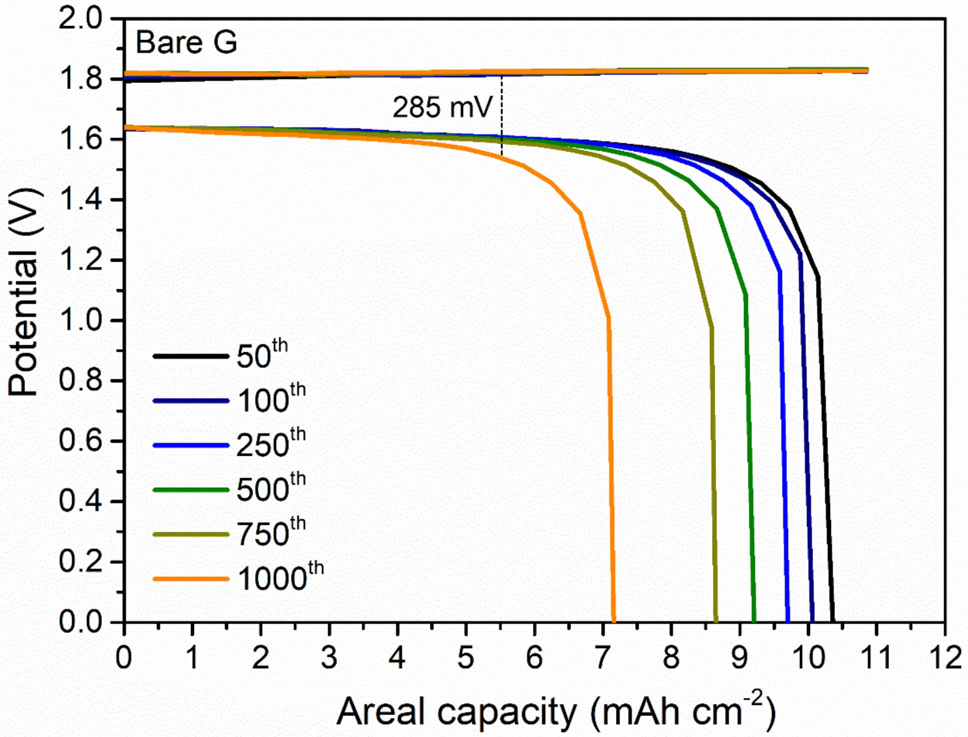

Figure S16. Voltage profile of the bare graphite at a current density of 25 mA cm^{-2} and an areal capacity of nearly 11 mAh cm^{-2}.

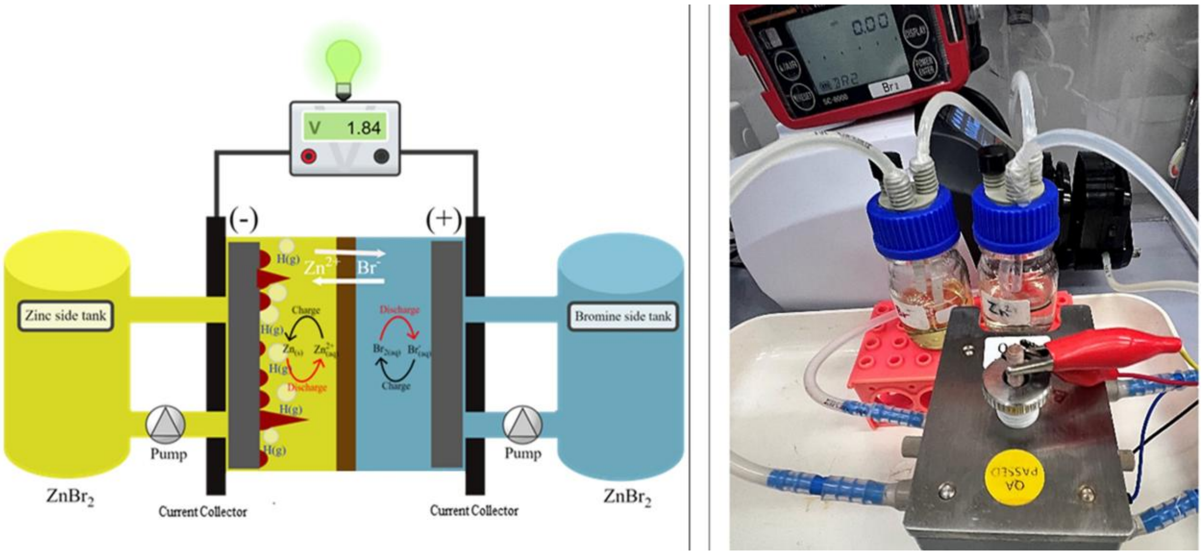

Figure S17. The zinc–bromine flow battery (1cm^2) used in this project, with a description image illustrating the cell configuration and working mechanism.

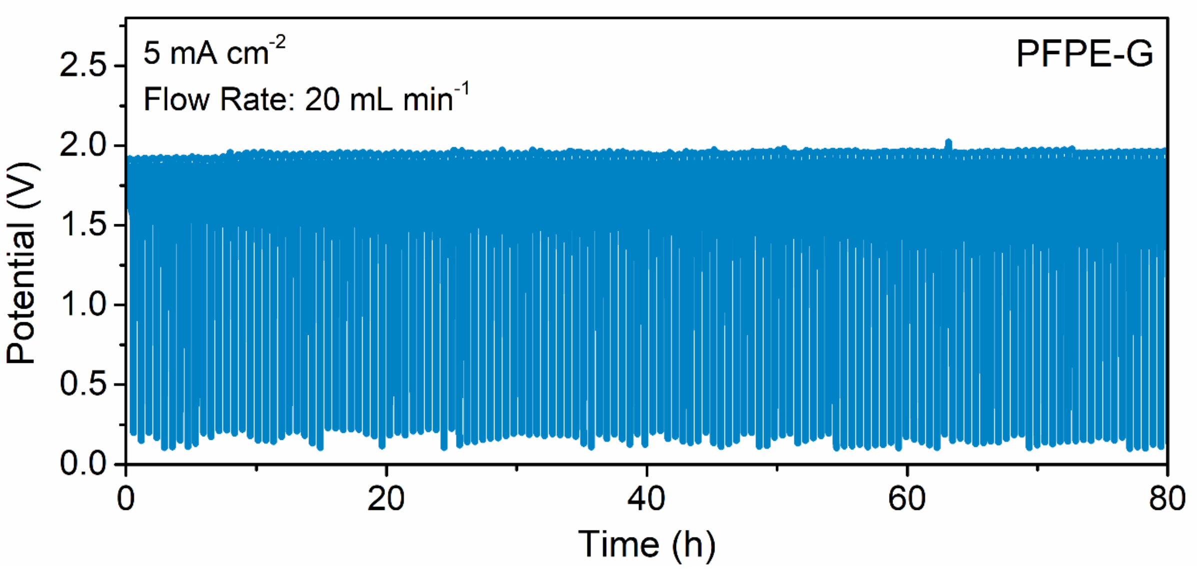

Figure S18. The cycling stability and performance of the zinc–bromine flow battery (1 cm^2) with the perfluoropolyether-coated graphite current collector under a current density of 5 mA cm^{-2}.

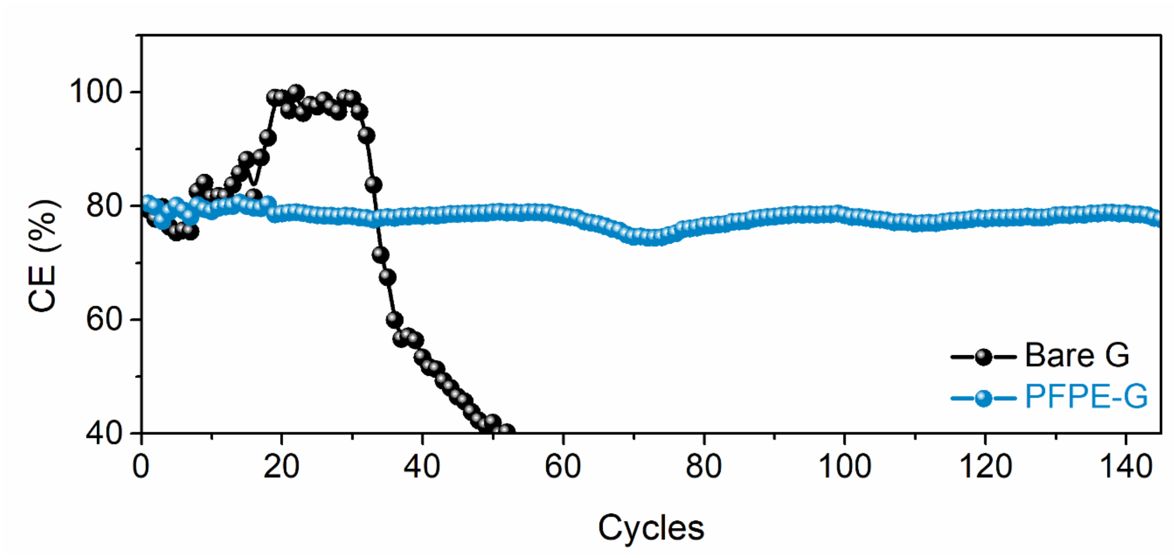

Figure S19. Coulombic efficiencies of the bare graphite and the perfluoropolyether-coated graphite zinc–bromine flow battery (1 cm^2) at 5 mA cm^{-2}.

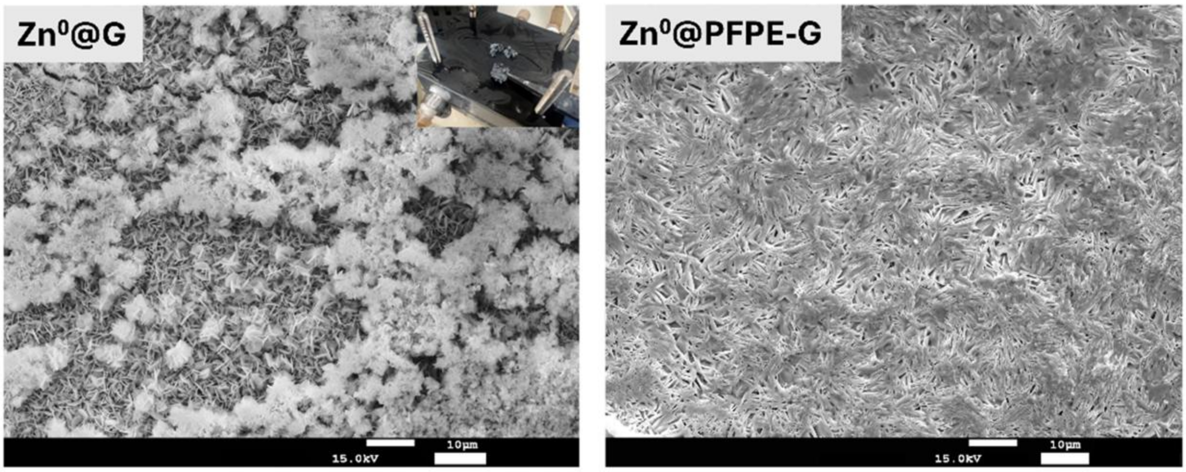

Figure S20. Scanning electron microscopy images illustrating the zinc-plating morphology on graphite (G; left) and perfluoropolyether-coated G (PFPE-G; right) current collectors in a zinc–bromine flow battery (ZBFB) after charging processes. Inset: Digital image of dead zinc plating observed after cycling in the ZBFB without the PFPE-G coating, indicating the formation of electrically isolated zinc due to uncontrolled deposition and poor interfacial stability.

Table S1. Cell performance parameters for the two non-flow ZBBs used in this study.

**Parameter**	**Cell 1**	**Cell 2**
Electrolyte concentration (M)	1.0	0.5
State of Charge (SoC, %)	15	40
Current (mA)	5	5
Current density (mA cm^{-2})	25	25
Areal capacity (mAh cm^{-2})	8.0	10.7
Energy density (Wh L^{-1})	14.4	19.3

Table S2. Energy-dispersive x-ray spectroscopy spectra with the percentage of the discharged perfluoropolyether-coated graphite samples (Fig. S11) before and after cycling.

Atomic ratio	Fluorine (%)	Zinc (%)
**Before cycling**	11.3	0.0
**After 1st cycle**	6.4	3.9

Table S3. Equivalent circuit element values used for fitting the electrochemical impedance spectroscopy spectra.

**Resistance (Ω)**	**Bare G**	**PFPE- G**
R_1	1.1	0.9
R_2	22.5	14.4
R_3	60.1	3.7

Table S4. Performance comparisons between the metrics of this study's battery and those of previously reported ZBBs.

Anode	Cathode Material	Electrolyte Composition	Additives	Separator	Battery Type	Areal capacity (mAh cm^{-2})	Current Density (mA cm^{-2})	Coulombic Efficiency (%)	Energy Efficiency (%)	Cycle Life (cycles)	Key Innovation	Reference
Graphite / PFPE-coated	Carbon felt	$ZnBr_2$ in water with additives	PFPE coating (forms ZnF_2 SEI layer)	Glass fiber	Static (non-flow) sandwich-type configuration	~11<br>8.0	25	>84<br>>94	>79<br>76	>1,000<br>>500	A hydrophobic PFPE interlayer enables in-situ formation of a ZnF_2-rich hybrid SEI for uniform Zn deposition, and suppression of HER	This Work
cation-exchange membrane (CEM) coating on Zn anode	N-defect decoration toward carbon felt cathode	$ZnBr_2$ in water	NA	*cation-exchange membrane coating*	Static (non-flow)	2	10	94.1	87.4	1,000	dual-interface strategy using a CEM-coated Zn anode and N-doped carbon cathode	ACS Appl. Mater. Interfaces, 2024[8]
Zn-coated Pt fabricated by electrodeposition methode	Protonated nitrogen (N)-doped microporous electrode	$ZnBr_2$ + HBr	No membranes, complexing agents, or supporting salts	None (membrane-free architecture)	Static (home-made with a 3D printer)	NA	NA	85	80	1,000	Multifunctional electrode for membrane-free Br_2 capture via polybromide confinement and conversion.	Adv. Mater., 2019[9]
Graphite felt	Graphite felt	$ZnBr_2$ + $CeCl_3$ in water	NMP-Br (cationic) + K^+ salt (anionic)	Glass fiber	Static (15 × 15 cm^2)	2	20	97.8	68.4	500	Dual ionic additives enhancing the reversibility of the zinc metal reaction	Small, 2024[10]
carbon cloth (CC)	Carbon foam electrode	$ZnBr_2$ + $ZnCl_2$ in water	None	None	Static (a single-chamber, membrane-free design)	NA	NA	~92	~60	1,000	Minimal architecture & cost-effective design	Energy Environ. Sci., 2017[11]
carbon cloth (CC)	carbon cloth (CC)	Zn-halide-based DES electrolyte	A deep eutectic solvent (DES)<br>BCM134 electrolyte.	Glass fiber	Static (coin-type configuration)	0.15	0.3	93	~60	900	DES electrolyte suppressing Br_2 loss	Adv. Sci., 2022[12]
Zn foil	nitrogen-doped carbon felt	$ZnBr_2$+alkaline KOH with glucose at the Zn anode, neutral KBr at the cathode for Br_2/Br^- redox	Glucose as an anolyte additive.	cation exchange membrane (CEM) of<br>polyethersulfone/sulfonated polyether ether ketone (PES/SPEEK-M)	Static (non-flow)	2	5	96.7	78	980	Decoupled Br_2/Zn pH regime for stability	Small Methods, 2024[13]
Zn foil	CMK-3 / Super P / PVDF	$ZnBr_2$ + TPABr in water	tetrapropylammonium bromide (TPABr) as the active material in the cathode	Glass fiber	Static (non-flow) sandwich-type configuration	6.3	1.27	99.9	94	11,000	Br_2 solid-phase trapping & Zn^{2+} plating aid	iScience, 2020[14]

References

1. Tao, S.; Zhang, C.; Zhang, J.; Jiao, Y.; Li, M.; Lin, W.; Ran, L.; Clement, B.; Lyu, M.; Gentle, I., A hydrophobic and fluorophilic coating layer for stable and reversible aqueous zinc metal anodes. *Chemical Engineering Journal* **2022,** *446*, 136607.
2. Huang, Y.; Wang, Y.; Peng, X.; Lin, T.; Huang, X.; Alghamdi, N. S.; Rana, M.; Chen, P.; Zhang, C.; Whittaker, A. K., Enhancing performance and longevity of solid-state zinc-iodine batteries with fluorine-rich solid electrolyte interphase. *Materials Futures* **2024**.
3. Rajarathnam, G. P.; Vassallo, A. M., *The Zinc/Bromine Flow Battery: Materials Challenges and Practical Solutions for Technology Advancement*. Springer: 2016.
4. Kresse, G.; Furthmüller, J., Efficiency of ab-initio total energy calculations for metals and semiconductors using a plane-wave basis set. *Comp. Mater. Sci.* **1996,** *6*, 15.
5. G. Kresse; Furthmüller, J., Efficient iterative schemes for ab initio total-energy calculations using a plane-wave basis set. *Phys. Rev. B* **1996,** *54*, 11169.
6. Perdew, J.; Burke, K.; Ernzerhof, M., Generalized Gradient Approximation Made Simple. *Phys. Rev. Lett.* **1996,** *77*, 3865.
7. Kondratowicz, I.; Nadolska, M.; Şahin, S.; Łapiński, M.; Prześniak-Welenc, M.; Sawczak, M.; Eileen, H. Y.; Sadowski, W.; Żelechowska, K., Tailoring properties of reduced graphene oxide by oxygen plasma treatment. *Applied Surface Science* **2018,** *440*, 651-659.
8. Liu, C.; Dong, W.; Zhou, H.; Li, J.; Du, H.; Ji, X.; Cheng, S., Achievement of Efficient and Stable Nonflow Zinc–Bromine Batteries Assisted by Rational Decoration upon the Two Electrodes. *ACS Applied Materials & Interfaces* **2024,** *16* (18), 23278-23287.
9. Lee, J. H.; Byun, Y.; Jeong, G. H.; Choi, C.; Kwen, J.; Kim, R.; Kim, I. H.; Kim, S. O.; Kim, H. T., High-Energy Efficiency Membraneless Flowless Zn–Br Battery: Utilizing the Electrochemical–Chemical Growth of Polybromides. *Advanced Materials* **2019,** *31* (52), 1904524.
10. Kim, J.; Park, H.; Cho, Y.; Lee, T.; Kim, H.; Pak, C.; Kim, H. J.; Kim, S., Stable Zinc Electrode Reaction Enabled by Combined Cationic and Anionic Electrolyte Additives for Non-Flow Aqueous Zn— Br2 Batteries. *Small* **2024**, 2401916.
11. Biswas, S.; Senju, A.; Mohr, R.; Hodson, T.; Karthikeyan, N.; Knehr, K. W.; Hsieh, A. G.; Yang, X.; Koel, B. E.; Steingart, D. A., Minimal architecture zinc–bromine battery for low cost electrochemical energy storage. *Energy & Environmental Science* **2017,** *10* (1), 114-120.
12. Heo, J.; Shin, K.; Kim, H. T., A Zinc–Bromine Battery with Deep Eutectic Electrolytes. *Advanced Science* **2022,** *9* (36), 2204908.
13. Dong, W.; Liu, C.; Tang, Z.; Cheng, S., Rational Design Toward Advanced Non-Flow Aqueous Zinc-Bromine Systems Boosted by Alkaline-Neutral Decoupling Electrolytes. *Small Methods* **2024,** *8* (12), 2400174.
14. Gao, L.; Li, Z.; Zou, Y.; Yin, S.; Peng, P.; Shao, Y.; Liang, X., A high-performance aqueous zinc-bromine static battery. *Iscience* **2020,** *23* (8), 101348.
